# Assessing the impact of health-care access on the severity of low back pain by country: a case study within the GBD framework

**DOI:** 10.1016/S2665-9913(24)00151-6

**Published:** 2024-07-16

**Authors:** YiFan Wu, Sarah Wulf Hanson, Garland Culbreth, Caroline Purcell, Peter Brooks, Jacek Kopec, Lyn March, Anthony D Woolf, Maja Pasovic, Erin Hamilton, Damian Santomauro, Theo Vos

**Affiliations:** a Institute for Health Metrics and Evaluation, University of Washington, Seattle, WA, USA; b Department of Biomedical and Health Informatics, University of Washington, Seattle, WA, USA; c Centre for Health Policy, School of Population and Global Health, University of Melbourne, VIC, Australia; d College of Health and Medicine, University of Tasmania, Hobart, TAS Australia; e School of Population and Public Health, University of British Columbia, Vancouver, BC, Canada; f Rheumatology and Musculosketal Epidemiology Medicine, Northern Clinical School, Sydney, NSW, Australia; g Royal Cornwall Hospitals Trust, Truro, UK; h Queensland Centre for Mental Health Research, Wacol, QLD, Australia; i School of Public Health, University of Queensland, Herston, QLD, Australia

## Abstract

**Background:**

The Global Burden of Diseases, Injuries, and Risk Factors Study (GBD) is key for policy making. Low back pain is the leading cause of disability in terms of years lived with disability (YLDs). Due to sparse data, a current limitation of GDB is that a uniform severity distribution is presumed based on 12-Item Short Form Health Survey scores derived from US Medical Expenditure Panel Surveys (MEPS). We present a novel approach to estimate the effect of exposure to health interventions on the severity of low back pain by country and over time.

**Methods:**

We extracted treatment effects for ten low back pain interventions from the Cochrane Database, combining these with coverage data from the MEPS to estimate the hypothetical severity in the absence of treatment in the USA. Severity across countries was then graded using the Health Access and Quality Index, allowing estimates of averted and avoidable burden under various treatment scenarios.

**Findings:**

We included 210 trials from 36 Cochrane systematic reviews in the network analysis. The pooled effect sizes (measured as a standardised mean difference) for the most effective intervention classes were –0·460 (95% uncertainty interval –0·606 to –0·309) for a combination of psychological and physical interventions and –0·366 (–0·525 to –0·207) for surgery. Globally, access to treatment averted an estimated 17·6% (14·8 to 23·8) of the low back pain burden in 2020. If all countries had provided access to treatment at a level estimated for Iceland with the highest Health Access and Quality Index score, an extra 9·1% (6·4 to 11·2) of the burden of low back pain could be avoided. Even with full coverage of optimal treatment, a large proportion (65·9% [56·9 to 70·4]) of the low back pain burden is unavoidable.

**Interpretation:**

This methodology fills an important shortcoming in the GBD by accounting for low back pain severity variations over time and between countries. Assumptions of unequal treatment access increased YLD estimates in resource-poor settings, with a modest decrease in countries with higher Health Access and Quality Index scores. Nonetheless, the large proportion of unavoidable burden indicates poor intervention efficacy. This method, applicable to other GBD conditions, provides policy makers with insights into health gains from improved treatment and underscores the importance of investing in research for new interventions.

**Funding:**

Bill and Melinda Gates Foundation and Queensland Health.

## Introduction

Low back pain is a highly prevalent condition worldwide. Most patients present with low back pain without an identifiable cause.^1^, ^2^, ^3^ A disc prolapse is an identified cause in a smaller subset of patients with back pain radiating to the legs; these patients usually experience worse symptoms and might require more invasive treatments like surgery.^4^, ^5^ From an economic perspective, low back pain is also known as a substantial burden to patients and societies due to work absenteeism and disability.^2^, ^6^

Low back pain is the largest contributor to years lived with disability (YLDs) in the Global Burden of Diseases, Injuries, and Risk Factors Study (GBD) 2021.^7^, ^8^ GBD is a comprehensive study incorporating information on mortality and morbidity to estimate the burden of 371 diseases and injuries systematically.^9^ YLDs, the non-fatal component of disability-adjusted life years (DALYs) are quantified as the product of prevalence, severity distributions, and disability weights for each severity or disease consequence level. Disability weights have been estimated in nine country surveys and an open-access internet survey. The main method used was pair-wise comparisons where respondents were asked “Who is the healthier” when presented with lay descriptions of two randomly chosen health states.^10^, ^11^ For low back pain, there are four health states for low back pain without leg pain (mild, moderate, severe, and most severe). The mild and moderate health states are also used for low back pain with leg pain, but the two most severe health states have a separate, higher disability weights for low back pain with leg involvement (appendix p 8). As there is a paucity of consistently measured data on the severity of low back pain across countries, the GBD uses an analysis of the Medical Expenditure Panel Surveys (MEPS), a large-scale overlapping continuous panel survey of the non-institutionalised US population primarily used to analyse the cost of health care. The same severity distribution is assumed to apply to all countries and time periods.^12^, ^13^ However, one would expect the burden of low back pain to vary depending on access and quality of health-care interventions.^14^ As a result, the current GBD method likely underestimates the burden of low back pain in countries with lower access to care than the USA.

**Research in context**

**Evidence before this study**

The current methods in the Global Burden of Diseases, Injuries, and Risk Factors Study (GBD) assume constant severity distribution of low back pain over time and between countries without considering the effect of health-care interventions. This is similarly the case for a large proportion of the top causes of years lived with disability (YLDs). Low back pain, because it is the top contributor to YLDs in GBD, was chosen as a case study to develop an alternative approach. A search of the Cochrane library on Aug 28, 2023, with the keywords “back pain” or “lumbar pain” or “backache” or “lumbago” identified 144 reviews. 36 reviews in addition to two randomised controlled trials representing 342 unique trials were included. The trials included a wide range of interventions for instance, physical interventions, exercises, and behavioural therapies.

**Added value of this study**

In this study, we present a novel approach to estimate the effect of exposure to health-care interventions on the severity of low back pain by country and over time. As a corollary, the analysis also allows quantification of currently averted and potentially avoidable burden with full access to optimal treatment. The method had been piloted for anxiety disorder and has the potential to be generalised to other non-fatal diseases in the GBD. The estimation of averted and avoidable burdens can serve as a benchmark for stakeholders to allocate resources to expanding intervention coverage and improving access to health-care interventions.

**Implication of all the available evidence**

The results showed that the burden in low-income and middle-income countries has been underestimated in the GBD, but also, that this higher burden is amenable to improved access to treatment. However, even if optimal treatment were to be fully implemented, most of the burden would remain. Our study emphasises the need for a comprehensive approach to low back pain and focus on primary prevention to manage and reduce the global burden of low back pain. Furthermore, it can function as a roadmap for stakeholders to turn GBD findings into actionable items if replicated across diseases.

The aim of this study is to present an alternative approach for estimating the burden of low back pain by considering the effects of health-care interventions on health state severity distribution. According to the 2023 WHO guideline for non-surgical management of chronic primary low back pain, various treatments, including physical interventions, psychological interventions, non-steroidal anti-inflammatory drugs, and multicomponent interventions are recommended depending on the condition of patients.^3^, ^15^, ^16^, ^17^ The proposed method also allows computation of the disease burden that is averted by current health-care interventions and would potentially be avoidable with full access to the recommended treatment.

## Method

### Study design

The study started with a meta-regression of the efficacy of ten classes of low back pain interventions, followed by quantifying their utilisation from the US MEPS. The hypothetical severity of low back pain without treatment was evaluated by removing the effect of reported treatments. The severity of low back pain across countries and time was linearly interpolated between a no-treatment scenario and the observed US treatment scenario in 2010, using the Health Access and Quality Index (appendix p 15). Finally, an optimal scenario was included, depicting full access to recommended treatments for each country and outlining the best possible outcome with current intervention knowledge (appendix p 4). This study follows the Guidelines for Accurate and Transparent Health Estimates Reporting statement (appendix p 3).^18^ We implemented this process as a Bayesian Markov chain Monte Carlo, taking into account 1000 samples from the posterior distribution at every stage of the estimation process, such as in the calculation of disability weights or the assessment of treatment effects. All the analyses were implemented in R version 3.6.0, and the 95% uncertainty intervals [UIs] were the 25th and 975th ranked values within the resulting distributions.

### Treatment effect estimation

Measures of the efficacy of health-care interventions for low back pain were obtained from the Cochrane Database of Systematic Reviews and two randomised controlled trials.^19^, ^20^ A review was conducted in the Cochrane Review Library on Aug 28, 2023, with the keywords “back pain” or “lumbar pain” or “backache” or “lumbago”. The exclusion criteria were: (1) treatments for conditions other than low back pain; (2) studies that did not explicitly review health-care interventions or treatments; (3) studies focusing on subpopulations like pregnant women and children; (4) reviews that only presented a qualitative analysis of the treatment effects; and (5) reviews that did not measure functional disability or status (appendix p 8). One researcher did the initial data extraction, and the second reviewer verified the initial extraction and added additional data. The researchers extracted the following items: intervention type, sample size and description, comparison type, measurement scale of functional status, outcome mean functional status and standard deviation, and follow-up duration. Interventions were grouped into ten classes: bed rest, education, epidural injection steroids, non-opioid analgesics, opioid analgesics, physical intervention, cognitive behavioural therapy (psychological), cognitive behavioural therapy combined with physical intervention, surgery, and a reference category of usual medical care or placebo treatment (appendix p 9). The follow-up time of all studies was restricted from 3 months to 12 months inclusive, and classes with fewer than ten studies were excluded.

The main outcome was the standardised mean difference (SMD) in functional disability, measured using different instruments including the Roland Morris Disability Questionnaire and Oswestry Disability Index, between an intervention and comparison group (appendix p 11). Functional disability was chosen as the main outcome because it correlates to the descriptions on which GBD disability weight were derived (appendix pp 4–7).^11^ SMDs from each trial were evaluated using the *escalc* function from R package metafor.^21^ The SMD expresses the difference in the change in score on a symptom scale between an intervention and comparator group as a fraction of the standard deviation of the scale. SMDs were pooled for all ten intervention classes using the network analysis function of a Bayesian, regularised mixed-effects meta-regression tool (MR-BRT) developed at the Institute for Health Metrics and Evaluation,^22^ which harmonises data with varying treatment and comparator groups (appendix pp 5, 9–10).

### Treatment use estimation

Treatment use estimates were acquired from MEPS responses to medical events through self-reported surveys in panels from 2000 to 2012, after which point the relevant survey questions were no longer included.^23^ The MEPS data were limited to respondents who self-reported having low back pain without leg pain (ICD-9 code 724, “other and unspecified disorders of back”) or with leg pain (ICD-9 code 722, “intervertebral disc disorders”; appendix pp 12–14).

### Severity estimation

The health state severity distribution of low back pain has been defined and quantified previously (appendix p 11).^12^ In GBD, the severity of low back pain is informed by MEPS data. Participants in MEPS completed the 12-Item Short Form Survey (SF-12), a short general health status questionnaire, twice over 2 years of observation (appendix pp 12–14). The SF-12 scores of each respondent were mapped to disability weights (appendix pp 4–7). To transform SF-12 summary scores into the scale of GBD disability weights, we used a convenience sample survey where respondents filled in SF-12 based on a subset of 62 out of the 231 health states for which disability weights were available across the spectrum of disability weight values.^24^ A meta-regression using MR-BRT was conducted, where the outcome was the logit-transformed disability weight, and the dependent variable was the SF-12 scores from MEPS (appendix pp 4–7). This gave an estimate of the cumulative disability weight of each individual after taking into account all other conditions they reported. To isolate the amount of disability weight that could be attributed to their low back pain in a regression with this cumulative individual disability weight as the logit transformed dependent variable and all diseases reported by MEPS respondents as the independent variables was run twice. By comparing the full model with a counterfactual in which we drop the term for low back pain, we isolated the amount of disability that can be assigned to low back pain as opposed to any comorbid other diseases. Lastly, the coverage of each intervention class was calculated as the proportion of low back pain patients across all panels utilising any of the ten intervention classes for which effect sizes were derived from the Cochrane reviews (appendix pp 13–14).

Next, a hypothetical scenario of greater severity in SF-12 terms for each respondent with low back pain if they had not received any intervention was estimated (equation 1) where in is the individual MEPS respondent

$$SF12_{no\;treatment,i}=SF12_{i}+SMD_{overall}\times \sigma_{SF12}$$

and SMD_overall_ the combined effect of any treatments reported by a respondent. If multiple intervention classes were reported by a respondent, the combined effect size was assumed to be multiplicative using equation 2.

$$SMD_{overall}=1-\prod_{i=\mathrm{int}ervention}(1-SMD_{i})$$

From the analysis of MEPS, we extrapolated a gradient of severity of low back pain in terms of expected disability weight by linearly interpolating the average disability weights in MEPS respondents and the hypothetical higher disability weight in the absence of any treatment using the Health Access and Quality Index.^25^ The Health Access and Quality Index is computed based on mortality from 32 causes amenable to personal health care and taken as an indicator of access and quality of health services in countries and over time. The no-treatment severity level was assumed to apply at the Health Access and Quality Index value 0. An average disability weight for each country and year was estimated by linearly interpolating from a Health Access and Quality Index value of 0 and disability weight assuming zero treatment, to the Health Access and Quality Index value of USA in 2007 (the midpoint for the available MEPS survey years) and the observed average disability weight in the MEPS data among people with low back pain (appendix pp 14–18).

Separately, in the full use of optimal treatment (FUOT) scenario, we assumed full use of the optimal treatment (psychological and physical interventions)^26^ and no sub-optimal interventions (equation 3).

$$SF12_{FUOT,i}=SF12_{no\;treatment,i}-SMD_{FUOT}\times \sigma_{SF12}$$

We assumed surgery was only indicated for those with low back pain and leg pain. According to a systematic review, a prolapsed disc spontaneously regresses in 57·1% of patients. We assumed the remainder (42·9%) of the patients would be eligible for surgical intervention.^4^ For those individuals with low back pain with leg pain eligible for surgical intervention, we applied the effect size of surgery to calculate the full use of optimal treatment scenario, and the above method applies to the remaining cases of low back pain with leg pain and those without leg pain.

### Averted burden and avoidable estimation

In addition, averted burden and avoidable burden were calculated. The averted burden was defined as YLDs that have been prevented through the existing health-care interventions by subtracting the current burden from the hypothetical no-treatment YLD estimate in each country. The avoidable burden with best routine care was estimated as the difference between YLDs for Iceland, the country with the highest Health Access and Quality Index value, and the current YLDs (equation 4). This assumes a

$$YLDS_{avoidable,BRC,ountry}=YLDS_{current,country}-LBP\;prev_{country}\times {\hat{DW}}_{Highest\;Health\;Access\;and\;Quality\;Index}$$

coverage of interventions for low back pain as observed in the MEPS population but extrapolated based on a country's Health Access and Quality Index value.

The avoidable burden with full use of optimal treatment scenario was estimated as the difference between the current burden and a hypothetical scenario in which everybody had full access to the most effective treatment from the meta-analysis. The values of averted, avoidable, and aspirational full use of optimal treatment burden have been computed for all countries, regions, super-regions, and globally (appendix pp 14–18).

### Role of the funding source

The funder of the study had no role in study design, data collection, data analysis, data interpretation or writing of the report.

## Results

A total of 144 Cochrane reviews were identified in the initial search of the Cochrane Systematic Reviews Database, and 36 reviews in addition to two randomised controlled trials, representing 210 unique trials were eligible for inclusion in the study (appendix pp 8, 10).^27^, ^28^, ^29^, ^30^, ^31^, ^32^, ^33^, ^34^, ^35^, ^36^, ^37^, ^38^, ^39^, ^40^, ^41^, ^42^, ^43^, ^44^, ^45^, ^46^, ^47^, ^48^, ^49^, ^50^, ^51^, ^52^, ^53^, ^54^, ^55^, ^56^, ^57^ The largest pooled effect size was a combination of psychological and physical interventions (SMD –0·460, 95% UI –0·606 to –0·309) followed by surgery (–0·366, –0·525 to –0·207; table 1).

**Table 1:** Intervention class standarised mean difference estimates and coverage estimates among respondents of the Medical Expenditure Panel Survey, 2007–12

	**Standarised mean difference (95% UI)**	**Coverage in low back pain with leg pain, % (95% UI)**	**Coverage in low back pain without leg pain, % (95% UI)**
Bed rest	0·141 (−0·0103 to 0·292)	Not surveyed	Not surveyed
Education	−0·109 (−0·211 to −0·0068)	Not surveyed	Not surveyed
Epidural injection steroids	−0·178 (−0·299 to −0·0570)	<0·1%	<0·1%
Non-opioid analgesics	−0·147 (−0·248 to −0·0467)	33·2% (31·8 to 34·7)	30·7% (30·0 to 31·4)
Opioid analgesics	−0·209 (−0·324 to −0·0952)	25·0% (23·7 to 26·4)	12·4% (11·9 to 12·9)
Physical intervention	−0·289 (−0·330 to −0·248)	30·6% (29·2 to 32·0)	20·6% (20·0 to 21·2)
Psychological and physical interventions	−0·460 (−0·606 to −0·309)	0·1% (0·0 to 0·2)	<0·1%
Psychological intervention	−0·255 (−0·401 to −0·109)	1·0% (0·7 to 1·3)	0·3% (0·3 to 0·4)
Surgery	−0·366 (−0·525 to −0·207)	10·1% (9·1 to 11·0)	2·0% (1·8 to 2·2)

UI=uncertainty interval.

For use of low back pain treatments in MEPS, the use rate of all interventions was higher in the group where leg pain was involved (appendix pp 13–14). The most used treatment was non-opioid analgesics in low back pain with leg pain (33·2% [95% UI 31·8 to 34·7]) and without leg pain (30·7% [30·0 to 31·4]), followed by physical intervention in low back pain with leg pain (30·6% [29·2 to 32·0]) and without leg pain (20·6% [20·0 to 21·2]). The MEPS survey-weighted combined SMD in low back pain with leg pain was –0·230 (–0·236 to –0·224), and without leg pain was –0·139 (–0·142 to –0·137).

Among MEPS respondents, the average disability weights were 0·169 (95% UI 0·168–0·169) for low back pain with leg pain and 0·103 (0·102–0·103) for low back pain without leg pain. The hypothetical no-treatment average disability weights were 0·228 (0·220–0·238) for low back pain with leg pain and 0·132 (0·129–0·137) for low back pain without leg pain. Under the full use of the optimal treatment scenario, the estimated disability weight was 0·143 (0·134–0·149) for patients with leg pain and 0·0776 (0·0701–0·0831) without leg pain (appendix p 17).

The location-specific severity-weighted disability weights derived for 21 GBD world regions are presented in table 2. Country-specific estimates are found in the appendix (appendix pp 19–23). The differences in mean disability weights for low back pain between the treatment-invariant weights used in GBD 2021 and the new treatment-specific disability weights were smallest in high-income regions with Health Access and Quality Index values similar to that of the USA during the years of MEPS data we used. The mean disability weight taking low access to treatment estimated in central sub-Saharan Africa was 16·7% higher than that estimated in GBD 2021. Computing global YLDs for low back pain with 6·2% higher mean disability weight would have changed the DALY ranking globally for low back pain from 8 to 5 in 2020. The country with the lowest location-specific weight caused by low back pain was Iceland at 0·092 (95% UI 0·063–0·127), and the country with the highest disability weight was the Central African Republic at 0·118 (0·079–0·171).

**Table 2:** Health Access and Quality Index values and disability weights used in GBD 2021 and those estimated for the year 2020 with a gradient on access to treatment globally, by GBD regions and super regions

		**Health Access and Quality Index**	**GBD 2021 disability weights**	**New disability weights**	**Disability weights change, %**
Global		55·6 (55·2 to 56·1)	0·096 (0·065 to 0·132)	0·102 (0·081 to 0·116)	6·2% (−15·5 to 42·0)
Central Europe, eastern Europe, and Central Asia		67·1 (66·4 to 67·8)	0·096 (0·065 to 0·132)	0·099 (0·080 to 0·113)	3·1% (−17·4 to 37·6)
	Central Asia	52·9 (52·1 to 53·9)	0·096 (0·065 to 0·132)	0·104 (0·083 to 0·119)	8·3% (−14·0 to 45·5)
	Central Europe	74·7 (73·8 to 75·4)	0·096 (0·065 to 0·132)	0·098 (0·079 to 0·111)	2·1% (−18·6 to 35·2)
	Eastern Europe	69·5 (68·2 to 70·7)	0·096 (0·065 to 0·132)	0·099 (0·080 to 0·112)	3·1% (−17·5 to 37·4)
High-income		85·8 (85·2 to 86·3)	0·096 (0·065 to 0·132)	0·095 (0·076 to 0·107)	−1·0% (−21·0 to 31·4)
	Australasia	90·7 (89·7 to 91·7)	0·096 (0·065 to 0·132)	0·093 (0·076 to 0·105)	−3·1% (−22·1 to 29·7)
	High-income Asia Pacific	89·0 (88·1 to 90·0)	0·096 (0·065 to 0·132)	0·094 (0·076 to 0·106)	−2·1% (−21·8 to 30·5)
	High-income North America	83·6 (82·0 to 84·9)	0·096 (0·065 to 0·132)	0·095 (0·077 to 0·108)	−1·0% (−20·5 to 32·2)
	Southern Latin America	64·6 (63·5 to 65·8)	0·096 (0·065 to 0·132)	0·100 (0·080 to 0·114)	4·2% (−16·5 to 39·6)
	Western Europe	89·2 (88·7 to 89·6)	0·096 (0·065 to 0·132)	0·094 (0·076 to 0·106)	−2·1% (−21·8 to 30·2)
North Africa and Middle East		57·3 (56·8 to 57·7)	0·096 (0·065 to 0·132)	0·102 (0·082 to 0·117)	6·2% (−15·0 to 43·3)
Sub–Saharan Africa		31·3 (30·7 to 31·7)	0·096 (0·065 to 0·132)	0·111 (0·088 to 0·130)	15·6% (−8·6 to 59·1)
	Central sub-Saharan Africa	28·3 (27·2 to 29·2)	0·096 (0·065 to 0·132)	0·112 (0·088 to 0·132)	16·7% (−8·0 to 61·2)
	Eastern sub-Saharan Africa	29·9 (29·3 to 30·5)	0·096 (0·065 to 0·132)	0·112 (0·088 to 0·131)	16·7% (−8·4 to 60·0)
	Southern sub-Saharan Africa	40·4 (39·1 to 42·0)	0·096 (0·065 to 0·132)	0·108 (0·085 to 0·126)	12·5% (−11·0 to 52·8)
	Western sub-Saharan Africa	31·8 (30·7 to 32·7)	0·096 (0·065 to 0·132)	0·111 (0·088 to 0·130)	15·6% (−8·5 to 58·7)
Latin America and Caribbean		53·6 (52·8 to 54·5)	0·096 (0·065 to 0·132)	0·104 (0·083 to 0·119)	8·3% (−14·1 to 45·5)
	Andean Latin America	55·3 (54·3 to 56·3)	0·096 (0·065 to 0·132)	0·103 (0·082 to 0·118)	7·3% (−14·3 to 44·6)
	Caribbean	47·9 (47·1 to 48·6)	0·096 (0·065 to 0·132)	0·105 (0·083 to 0·121)	9·4% (−13·2 to 47·8)
	Central Latin America	54·5 (53·5 to 55·8)	0·096 (0·065 to 0·132)	0·103 (0·082 to 0·118)	7·3% (−14·2 to 44·6)
	Tropical Latin America	53·3 (52·0 to 55·4)	0·096 (0·065 to 0·132)	0·104 (0·083 to 0·120)	8·3% (−14·0 to 45·5)
South Asia		39·4 (37·9 to 41·1)	0·096 (0·065 to 0·132)	0·108 (0·086 to 0·126)	12·5% (−10·5 to 53·3)
Southeast Asia, east Asia, and Oceania		64·4 (63·4 to 65·3)	0·096 (0·065 to 0·132)	0·100 (0·080 to 0·114)	4·2% (−16·4 to 39·5)
	East Asia	72·9 (71·3 to 74·2)	0·096 (0·065 to 0·132)	0·098 (0·079 to 0·111)	2·1% (−18·0 to 36·0)
	Oceania	31·6 (29·6 to 33·2)	0·096 (0·065 to 0·132)	0·111 (0·087 to 0·130)	15·6% (−8·9 to 58·5)
	Southeast Asia	47·1 (46·0 to 47·9)	0·096 (0·065 to 0·132)	0·106 (0·084 to 0·122)	10·4% (−12·6 to 49·5)

Data in parentheses are 95% uncertainty intervals. GBD=Global Burden of Diseases, Injuries, and Risk Factors Study.

Globally, 17·6% (95% UI 14·8–23·8) of low back pain in 2020 would have been averted if all countries had the lowest disability weight value, as computed for Iceland, the country with the highest Health Access and Quality Index value in 2020. This can be considered the avoidable burden if all countries had access to routine health care like Iceland. Under the full use of the optimal treatment scenario, 9·1% (6·4–11·2) could be avoided (figure, appendix pp 24–27). However, full access to the most effective treatments would only be able to reduce the untreated burden by 7·4% (6·4–9·1), leaving most of the burden unavoidable (65·9% [56·9–70·4]; figure).

**Figure:**
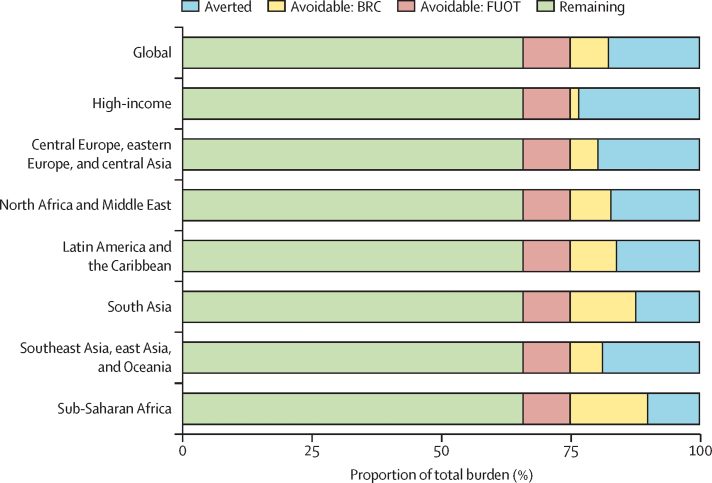
The proportion of low back pain burden that is averted by current treatment, could be averted with BRC or FUOT, and remaining burden that is not amenable to treatment, globally and by GBD super-regions, 2020 BRC=best routine care (scenario). FUOT=full use of the optimal treatment (scenario). GBD=Global Burden of Diseases, Injuries, and Risk Factors Study. High-income category included countries from seven GBD super-regions.

## Discussion

In line with the aim of this study, the proposed method to approximate a gradient of severity of low back pain by country incorporates various sources of information, including Cochrane reviews, use of interventions, Health Access and Quality Index, and disability data from GBD. This approach provides a more plausible distribution of the disability from low back pain across geographies than assuming the same severity distribution applied to all locations. By accounting for the variability in severity based on health-care use and outcomes, this method offers a more nuanced understanding of the burden of low back pain worldwide.

We examined all treatment interventions evaluated in the Cochrane reviews of low back pain. According to the 2023 WHO guidelines for the non-surgical management of chronic low back pain, various treatment regimens are advised based on patient conditions. The guidelines recommend that patients remain physically active, and conditionally recommend multicomponent therapies, which can include combinations of psychological and physical therapies, depending on the specifics of each individual.^3^ Our results align with the guidelines that the most efficacious treatment for patients without leg pain is an integrated therapeutic approach combining psychological therapies and physical interventions. For patients with leg pain, surgical intervention is the most effective treatment for those with a prolapsed or herniated disc that does not spontaneously remit. In our analysis, there were few studies using combinations of physical interventions, psychological interventions, and non-opioid analgesics as the treatment, and we were unable to calculate a pooled effect size. Therefore, we chose the effect size for physical intervention along with psychological treatment (the greatest effect size) for the FUOT treatment. It is important to emphasise that physical and psychological therapies are often the most effective, yet they are also more challenging to access and require more resources to provide.^58^ Enhancing their availability necessitates prioritising them at the level of health system planning. According to the literature, the use of non-opioid analgesics for both acute and chronic low back pain is recommended in most of the low back pain clinical guidelines across the world.^26^, ^59^ In our study, opioid pain medications were not nearly as effective as psychological and physical interventions, providing an argument for an opioid-sparing approach to pain management in addition to the problems of addiction and consequent major threats to health.^59^, ^60^

Applying treatment varying disability weights to the GBD 2021 results for low back pain would have resulted in 6·2% higher estimates of YLDs, ranging from a –3·1% decrease in Australasia to a 16·7% increase in central sub-Saharan Africa and eastern sub-Saharan Africa. Even in the FUOT scenario, most of the burden remains because a large proportion of the low back pain burden cannot be alleviated using existing health-care technologies. This highlights the importance of developing new treatment approaches to address the burden of low back pain further. This study addresses an important issue and criticism^61^ that low back pain severity distributions are assumed to be constant over time and location under the current GBD framework.^14^

There are several limitations to this study. First, we pooled data from trials identified by systematic reviews from the Cochrane Library with varying design quality and definition of interventions, making it challenging to harmonise data.^62^ The Bayesian meta-regression method incorporates unexplained between-trial heterogeneity into the uncertainty ranges of pooled effect sizes, which can partially reflect the additional error from pooling heterogeneous studies. Moreover, MEPS responses are based on self-report which could introduce reporting bias. In MEPS, it was found that self-reported hospitalisations were accurate but emergency department visits and office visits were under-reported by a third and 19%, respectively.^63^ Second, classes of interventions in the analysis were subjectively constructed, with interventions assigned to the most appropriate class according to the authors' discretion. For instance, the physical intervention group contains a broad spectrum of interventions, including active physical interventions like exercise, and passive physical interventions like massage and ultrasound. We wanted to use more granular classes, but some did not have enough trials to calculate reliable effect sizes. This could have affected the results of the analysis. Third, we presumed a pooled SMD from diverse symptom scales could apply to SF-12 measurements. This assumption, fundamental in meta-analyses of treatment effect sizes expressed on a symptom scale, is valid when scales are correlated. However, the few studies reporting on correlations between SF-12 scores and physical functioning scales found only a moderate correlation.^64^, ^65^ Fourth, the Health Access and Quality Index was derived from the mortality rate of 32 causes of death that are deemed preventable using effective health care.^25^ It is the best instrument available to assess health-care accessibility and quality. However, it only approximates what true variations might be in the coverage of treatments of low back pain between countries. Fifth, there was no involvement of people with lived experience as we performed a secondary analysis of existing data sources. Last, GBD estimates for low back pain prevalence account for individuals reporting low back pain on the day of the survey, which captures both short-duration episodes and more chronic conditions. Our intervention impact data specifically pertains to those with more chronic manifestations of low back pain. Consequently, our coverage estimates imply that individuals with short-duration episodes are less likely to receive interventions of interest, aside from pain relief medications. This approach partly explains the relatively low coverage of many interventions, considering that those with chronic low back pain represent only a subset of all individuals with low back pain. We acknowledge that there might be an overestimation of coverage, which is derived from MEPS data, and reflects only those individuals that have sought some form of health care.

Despite these limitations, the proposed approach is an improvement over the current practice of assuming the same severity of low back pain regardless of health care intervention. This method serves as a preliminary step in re-examining the estimation of disability weights. If, in due time, the severity of low back pain is measured more frequently and consistently, we can compare these new results with directly observed data. Also, if data on access to treatments are more systematically collated, directly observed treatment proportions can be built into this method rather than the coarse interpolation along the scale of Health Access and Quality Index. As such, changes in low back pain burden reported in future GBD studies may reflect methodological changes rather than actual epidemiological shifts. We can accompany the reporting of future low back pain burden estimates with a decomposition of the impact of new data, changed severity distributions, and other methodological changes.

We are committed to further validating and refining these methods through future research. The method can be generalised to other non-fatal diseases and conditions whose severity distributions are not well characterised. For instance, a similar analysis of a gradient in the severity of anxiety disorders has recently been conducted and suggests that the same methods can be applied to other diseases in the GBD for which currently non-variant severity distributions are assumed between locations and over time.^66^ In addition, this new method allowed us to approximate the currently averted, avoidable, and remaining burden, which is very interpretable and actionable. This work has the potential to be coupled with an estimation of the cost-effectiveness of intervention packages to guide resource allocation.

In light of our findings that a substantial portion of the low back pain burden remains unavoidable even with optimal treatment access, it becomes imperative to expand our focus beyond treatment efficacy to include prevention and public education.^1^ Implementing strategies like exercise alone or in combination with education has shown effectiveness in preventing low back pain,^67^, ^68^ whereas early interventions and comprehensive rehabilitation programmes can mitigate its progression.^1^, ^3^ Normalising low back pain as a manageable condition encourages timely care seeking and offers a holistic approach to low back pain care combining the knowledge of patient education and self-management with regular medical treatments, potentially reducing the global impact of low back pain.

### Contributors

### Data sharing

All input data extractions of treatment effects are available upon publication in https://github.com/yifwu/Low_Back_Pain_Burden_Estimation. Sequela-level GBD estimates of low back pain prevalence by severity are available upon request up to 1 year after publication; requests should be directed to Sarah Wulf Hanson at swulf@uw.edu.

## Declaration of interests

We declare no competing interests.
