# Supplementary material for: Assessing the impact of health-care access on the severity of low back pain by country: a case study within the GBD framework
Source: Lancet Rheumatol. 2024 Jul 16;6(9):e598–606. doi: 10.1016/S2665-9913(24)00151-6 (PMC11333387; doi:10.1016/S2665-9913(24)00151-6)
Supplement: Supplementary appendix [file mmc1.pdf]

THE LANCET
Rheumatology

Supplementary appendix

This appendix formed part of the original submission and has been peer reviewed. We post it as supplied by the authors.

Supplement to: Wu Y, Wulf Hanson S, Culbret G, et al. Assessing the impact of health-care access on the severity of low back pain by country: a case study within the GBD framework. *Lancet Rheumatol* 2024; published online July 16. https://doi.org/10.1016/S2665-9913(24)00151-6.

Supplementary Material: Assessing the impact of healthcare access on the severity of low back pain by country

Table of Contents

Table S1: Guidelines for Accurate and Transparent Health Estimates Reporting (GATHER) checklist 3

Figure S1. A conceptual overview of the data analysis method. 4

Section 1. Mapping 12-item Short Form Health Surveys to disability weight. Improvements made compared to the other methods 4

Figure S2 Regression of individual-level SF-12 scores on disability weights via quadratic spline in MR-BRT. 5

Figure S3: Regression of aggregate-level SF-12 scores on disability weights via quadratic spline in MR-BRT. 6

Figure S4 Regression of individual-level SF-12 scores on disability weights via quadratic spline in MR-BRT using spline priors from the aggregate model. 7

Section 2: Extraction of Cochrane reviews and Prism diagram 7

Figure S5. PRISMA diagram for the search of Cochrane library for reviews of LBP treatment effects. 8

Table S2. Classification of treatment classes and control classes(opioid analgesics and epidural injection steroids are stand alone categories). 9

Table S3. Breakdown of active physio interventions and passive physio interventions. 9

Table S3. Network meta-analysis comparison counts. The number of trials for each intervention reference group pair in the pooled effect size estimation. 10

Table S4. The most frequently used instruments measuring disability in the network analysis(only the top 6 are shown). 11

Table S5. The current definition for the eight low back pain health states and their latest disability weight with 95% uncertainty interval. 11

Table S6. Results compared against WHO non-surgical guidelines. 11

Section 3: Estimation of LBP in current MEPS data 12

Figure S6. Utilization plots using MEPS data by MEPS panel. Shaded areas represent a 95% confidence interval. Top: Individual treatment; Bottom: combined treatment. 13

Section 4: Grading severity by healthcare access 14

Figure S7. Adjustment factor to 2007 US Medical Expenditure Panel Survey sequela-weighted disability weight varying by healthcare access quality index 15

Figure S8. MEPS disability weight distributions by the scenario in the MEPS sample for LBP with and without leg involvement. Density plots of three scenarios: 1) with no treatment 2) with current treatment 3) full utilization optimal treatment. Black lines represent the mean of the distribution. 17

Figure S9. Distribution of disability weights among LBP cases for three scenarios (Upper Fig: LBP without leg pain; Lower Fig: LBP with leg pain. Dotted lines represent the cutoffs) in MEPS. 18

Table S6. Health Access and Quality index (HAQi) values and disability weights used in GBD 2021 and those estimated for the year 2020 with a gradient on access to treatment by country, region, and super-region 19

Table S7: Predicted severity proportions for low back pain for 2020 by country, region and super-region using the data with current treatment **24**

Table S8. PRISMA Checklist **28**

References........ **32**

Table S1: Guidelines for Accurate and Transparent Health Estimates Reporting (GATHER) checklist

Item #	Checklist item	Reported on page #
**Objectives and funding**		
**1**	Define the indicator(s), populations (including age, sex, and geographic entities), and time period(s) for which estimates were made.	Methods (pages 5-8)
**2**	List the funding sources for the work.	Financial support (pages 2, 14)
**Data Inputs**		
For all data inputs from multiple sources that are synthesized as part of the study:		
**3**	Describe how the data were identified and how the data were accessed.	Methods (pages 5-6)
**4**	Specify the inclusion and exclusion criteria. Identify all ad-hoc exclusions.	Methods (page 5) and appendix (pages 7-8)
**5**	Provide information on all included data sources and their main characteristics. For each data source used, report reference information or contact name/institution, population represented, data collection method, year(s) of data collection, sex and age range, diagnostic criteria or measurement method, and sample size, as relevant.	*https://github.com/yifwu/Low_Back_Pain_Burden_Estimation*
**6**	Identify and describe any categories of input data that have potentially important biases (e.g., based on characteristics listed in item 5).	Methods (pages 5-6) and appendix (pages 4-6 MR-BRT)
For data inputs that contribute to the analysis but were not synthesized as part of the study:		
**7**	Describe and give sources for any other data inputs.	Methods (page 5-6), appendix (pages 4-5)
For all data inputs:		
**8**	Provide all data inputs in a file format from which data can be efficiently extracted (e.g., a spreadsheet rather than a PDF), including all relevant meta-data listed in item 5. For any data inputs that cannot be shared because of ethical or legal reasons, such as third-party ownership, provide a contact name or the name of the institution that retains the right to the data.	*https://github.com/yifwu/Low_Back_Pain_Burden_Estimation*
**Data analysis**		
**9**	Provide a conceptual overview of the data analysis method. A diagram may be helpful.	Figure S1 (page 4)
**10**	Provide a detailed description of all steps of the analysis, including mathematical formulae. This description should cover, as relevant, data cleaning, data pre-processing, data adjustments and weighting of data sources, and mathematical or statistical model(s).	Methods (pages 4-8 and appendix (pages 14-17)
**11**	Describe how candidate models were evaluated and how the final model(s) were selected.	Methods (pages 4-7) and appendix (pages 5-6)
**12**	Provide the results of an evaluation of model performance, if done, as well as the results of any relevant sensitivity analysis.	N/A

**13**	Describe methods for calculating uncertainty of the estimates. State which sources of uncertainty were, and were not, accounted for in the uncertainty analysis.	Methods (pages 5-8)
**14**	State how analytic or statistical source code used to generate estimates can be accessed.	*https://github.com/yifwu/Low_Back_Pain_Burden_Estimation*
**Results and Discussion**		
**15**	Provide published estimates in a file format from which data can be efficiently extracted.	Appendix (Table S7, Table S8)
**16**	Report a quantitative measure of the uncertainty of the estimates (e.g. uncertainty intervals).	*All estimates are provided with 95% uncertainty intervals.*
**17**	Interpret results in light of existing evidence. If updating a previous set of estimates, describe the reasons for changes in estimates.	Discussion (pages 11-13)
**18**	Discuss limitations of the estimates. Include a discussion of any modelling assumptions or data limitations that affect interpretation of the estimates.	Discussion (pages 13-14)

Figure S1. A conceptual overview of the data analysis method.

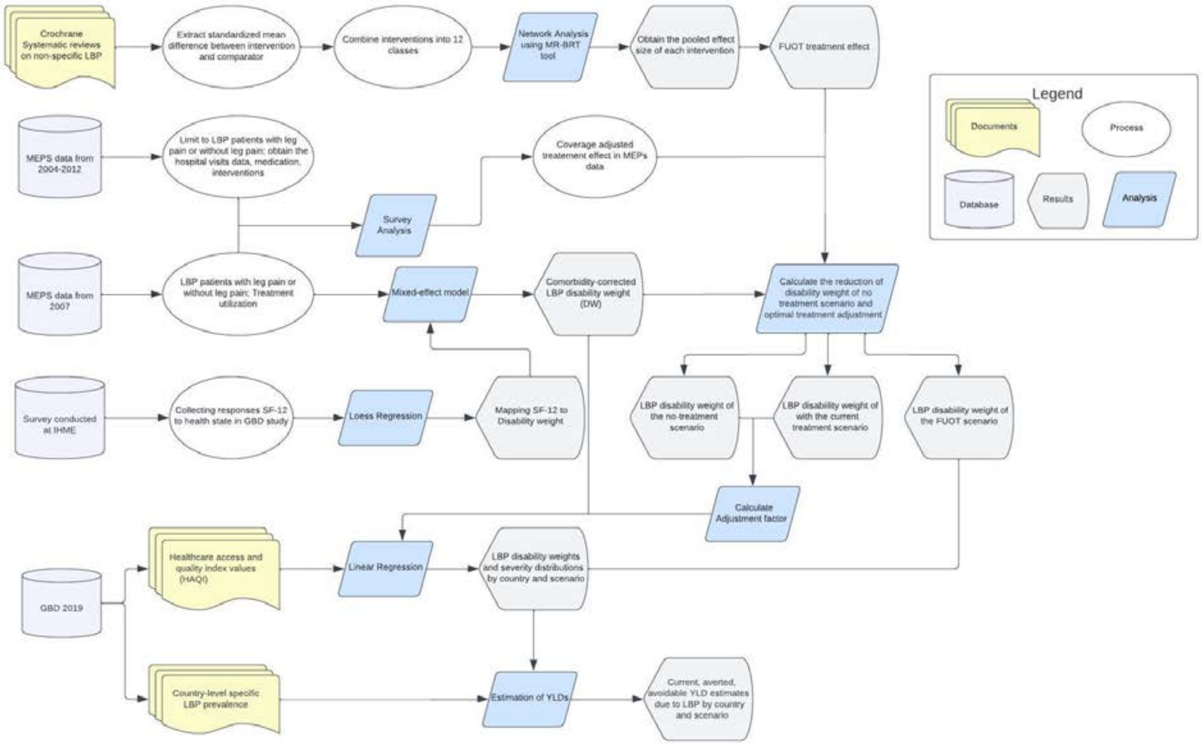

Section 1. Mapping 12-item Short Form Health Surveys to disability weight. Improvements made compared to the other methods

For GBD 2013, a 12-item Short Form Health Survey with questions asking about hypothetical individuals experiencing specific health states was conducted among 2338 respondents providing SF-12 scores for 62 health

states[1]. Each respondent completed Sf-12 forms with up to 50 randomly selected health states. These health states were randomly selected from a section with 62 health states in GBD that reflected a broad range of spectrum of health state DWs from least to most severe[1]. This map derived from this study was used in all subsequent GBD studies.

In the original method, due to the high heterogeneity among SF-12 scores, SF-12 scores with more than two absolute deviations from the median, approximately 18% of the observations, were removed from the analysis. A loess model was conducted on the observed SF-12 score for each health state and its average disability weight to obtain the estimated SF-12 score for each health state. The model was adjusted further with a mixed effect model.

The original method is straightforward but also comes with some deficiencies. The exclusion method was too crude and about 1/5 of the observations were removed. With a more refined method, more observations could be retained in the analysis. Secondly, no transformation was used before the loess model so that the mean disability weight could be lower than 0 or greater than 1, which does not align with the current method. Moreover, the loess model does not include uncertainty, and the original method used average disability weight in the model instead of individual response.

The new method addresses all the issues listed above. First, a logit transformation was used to solve the issues that the disability weight is beyond the range from [0,1]. We conducted a meta-regression using SF-12 scores and the transformed disability weight via a quadratic spine using MR-BRT (Meta-Regression: Bayesian, Regularized, Trimmed)[2]. For the first improvement, instead of using median, we used SF-12 scores ± 1.64* Standard deviation from the mean representing the top and bottom 5% were removed. About 9% of the observation were excluded in the end. We started using SF-12 scores on disability weights via quadratic spline, but the spline failed because a substantial number of observations for health states had a disability weight of less than 0.5 (Figure S2). We modified the first model by using aggregated data instead of using data with an individual health state, which improved from the first model across the entire spectrum (Figure S3). However, the aggregated SF-12 scores would ignore the heterogeneity of the individual observations. Therefore, we finalized our model with individual level SF-12 scores and used the intercept of the spline coefficients from the aggregate model as a prior method, and included gamma in the prediction to obtain uncertainty. The final model covers a broad spectrum of disability weight with uncertainty (Figure S4).

MR-BRT

MR-BRT(meta-regression- Bayesian, regularized, trimmed) was initially developed in-house at IHME and this tool has been applied widely in recent GBD studies. It is a customized meta-regression tool and the model is a trimmed constrained mixed-effect model that solves common linear and nonlinear mixed effects models using maximum likelihood estimation[2].

Since we use MR-BRT for the meta analysis on the Cochrane reviews, to account for study heterogeneity, a gamma term(with a precision of 0.001) and random effects were used. The Gamma term is the coefficient of SMD. Since the precision is 0.001, this would suggest the standard error of the gamma coefficient is very low, indicating high confidence in the stability and reliability of this estimate within the meta regression model. We used the 10% trimming option, and we removed the top 10% outliers in the meta-regression analysis to ensure heterogeneity.

MR-BRT is open source and the package is available on Github:
https://github.com/ihmeuw-msca/xspline
https://github.com/zhengp0/limetr

MRTool Github Repository:
https://github.com/ihmeuw-msca/mrtool

Figure S2 Regression of individual-level SF-12 scores on disability weights via quadratic spline in MR-BRT.

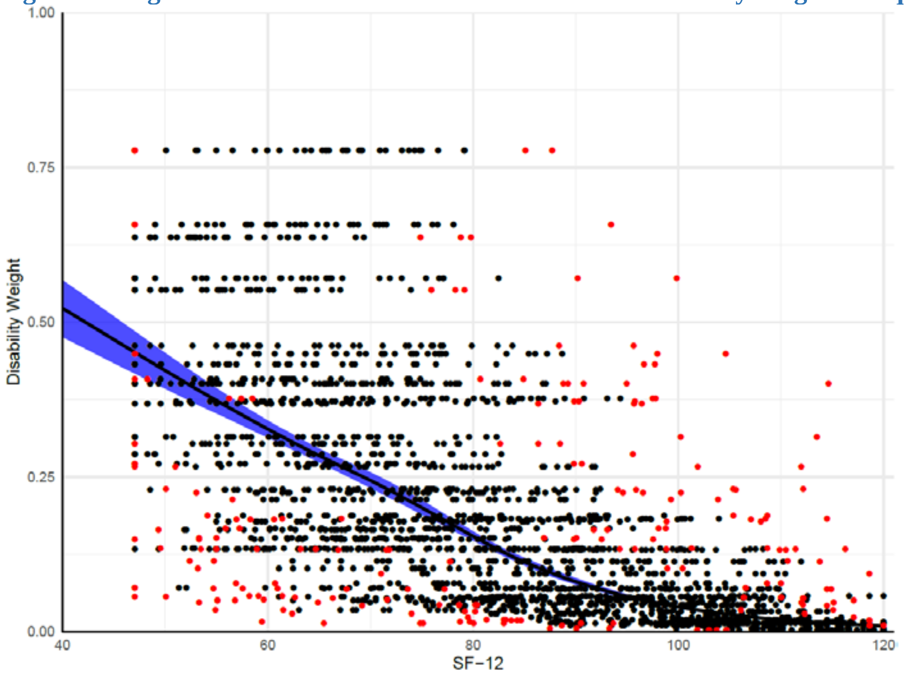

Black points = SF-12 scores. Red points = Outliers. SF-12 scores. SF-12 = 12-item Short Form Health Survey.

Figure S3: Regression of aggregate-level SF-12 scores on disability weights via quadratic spline in MR-BRT

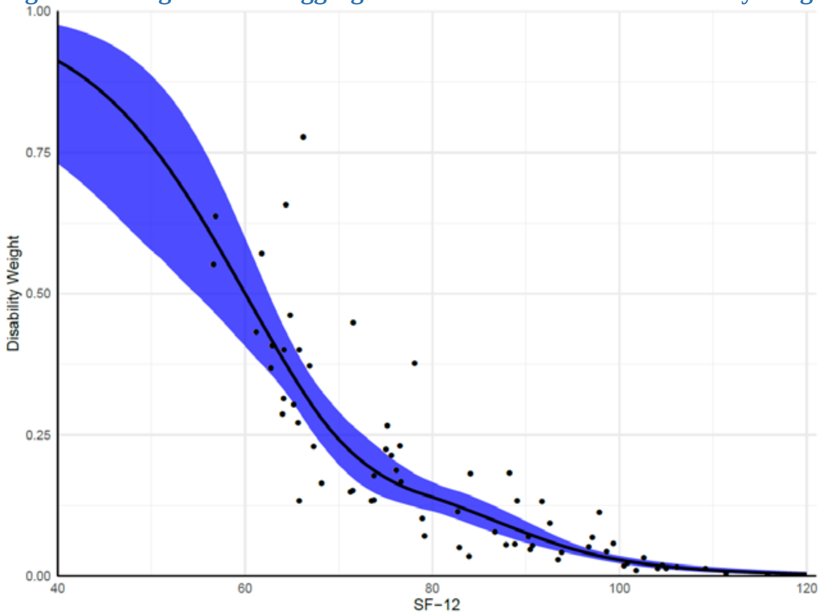

Black points = Average SF-12 scores by health state. SF-12 = 12-item Short Form Health Survey.

Figure S4 Regression of individual-level SF-12 scores on disability weights via quadratic spline in MR-BRT using spline priors from the aggregate model.

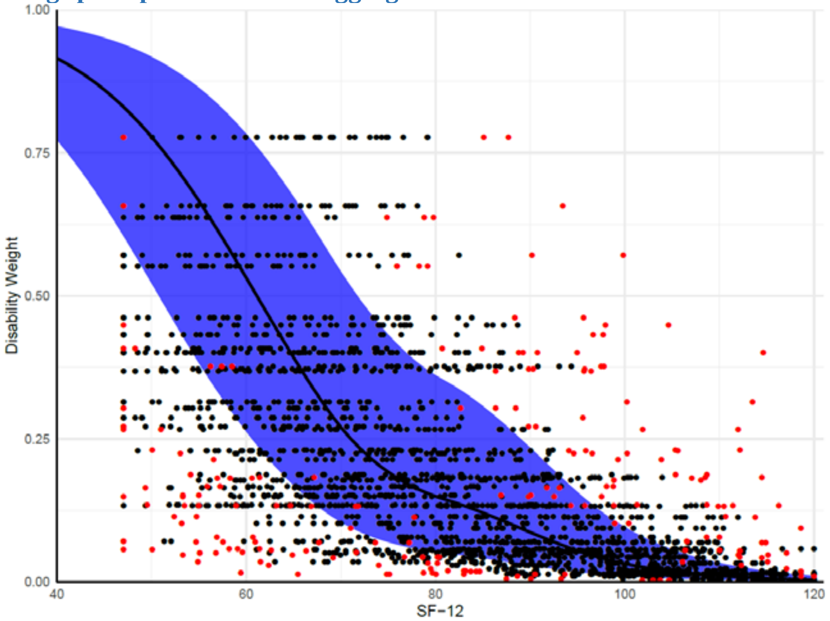

Black points = SF-12 scores. Red points = Outliered SF-12 scores. SF-12 = 12-item Short Form Health Survey.

Section 2: Extraction of Cochrane reviews and Prism diagram

The prism diagram depicts the inclusion and exclusion criteria (Figure S5). For the extraction, the initial extraction was done by a researcher independently. A second researcher did a second pass later to ensure the inclusion of papers was valid. Several items were extracted: intervention description, intervention type, comparison description, comparison type, measurement scale, follow-up duration, intervention and comparison sample sizes, outcome mean, and outcome SD. Studies that had repetitive information or non-reproducible standard mean difference were removed.

Figure S5. PRISMA diagram for the search of Cochrane library for reviews of LBP treatment effects. The exclusion criteria were: 1) treatments for conditions other than LBP 2) studies that did not explicitly review healthcare interventions or treatments, for example, review of diagnostic criteria, and prescription adherence 3) studies focusing on subpopulations like pregnant women and children 4) reviews that only presented a qualitative analysis of the treatment effects 5) reviews that did not measure functional disability or status. In addition, we included reviews that measure the treatment effect of LBP vs. the effect in the reference group on a measure of functional status and disability.

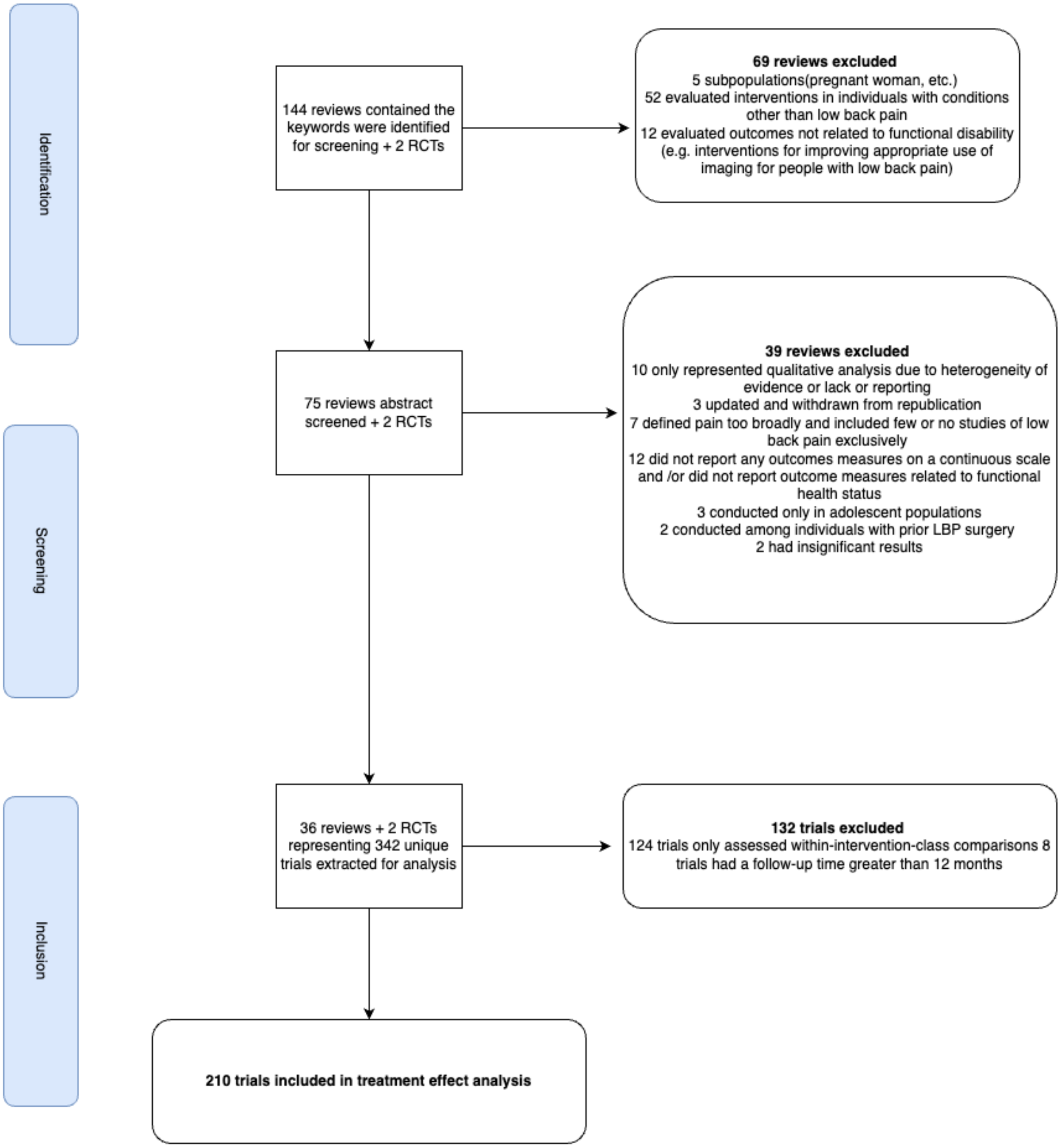

Table S2. Classification of treatment classes and control classes(opioid analgesics and epidural injection steroids are stand alone categories).

Classified Class	Original Class
Physical Interventions	Active physio + passive physio
	Active physio + education
	Active physio + education + psychological
	Active physio
	Passive physio
	Active physio + passive physio + education
	Passive physio + education
	Active physio + Placebo (control class only)
	Passive physio + Placebo (control class only)
	Passive physio + Care as usual (control class only)
Psychological and Physical Interventions	Active physio + passive physio + education + psychological
	Active physio + passive physio + psychological
	Passive physio + psychological
	Active physio + psychological
Psychological	Psychological + Passive physio + psychological
	Psychological + educationActive physio + psychological
Psychological therapies, Physical Interventions and Non-opioid analgesics	Care as usual + Active physio + education + psychological + pharmacological therapy – non-opioid
	Placebo + Active physio + passive physio + education + psychological +pharmacological therapy – non-opioid
Psychological	Care as usual + education + Psychological
	Wait list control + Psychological + education
Control	Care as usual
	Placebo
	Care as usual + education
	Wait list control

Table S3a. Breakdown of active physio interventions and passive physio interventions.

Classified Class	Original Class
Active Physio Intervention	Exercise
	Yoga
	Yoga + Exercise
	Motor Control Exercise
	Pilates
	Aerobic exercise
Passive Physio Intervention	Therapeutic ultrasound
	Muscle energy technique
	Heat therapy
	Acupuncture
	Acupuncture + standard therapy
	Massage
	C-TENS
	A-TENS
	Chiropractic
	Traction
	Low level laser therapy
	Physiotherapy with traction

Table S3b. Network meta-analysis comparison counts. The number of trials for each intervention reference group pair in the pooled effect size estimation.

Intervention class	Comparison class	Number of trials
Physical Intervention	Control	227
Non-opioid analgesics	Control	26
Epidural injection steroids	Control	22
Physical interventions	Education	26
Surgery	Control	14
Psychological	Control	15
Opioid analgesics	Control	12
Bed Rest	Control	9
Psychological and Physical interventions	Physical interventions	13
Education	Physical interventions	7
Education	Control	6
Physical interventions	Non-opioid analgesics	6
Psychological	Physical interventions	6
Bed Rest	Physical interventions	4
Physical interventions	Psychological	3
Physical interventions	Physical interventions and Non-opioid analgesics	2
Physical interventions	Bed Rest	2
Psychological	Surgery	2
Opioid analgesics	Non-opioid analgesics	1
Psychological therapies and Non-opioid analgesics	Control	1
Surgery	Epidural injection steroids	1
Surgery	Non-opioid analgesics	1

Table S4. The most frequently used instruments measuring disability in the network analysis(only the top 6 are shown).

Instrument	Number of trials
Roland Morris Disability Questionnaire	167
Oswestry Disability Index	124
Sickness Impact Profile	26
Patient-Specific Functional Scale	12
Pain Disability Index	9
Quebec Disability Scale	7

Table S5. The current definition for the eight low back pain health states and their latest disability weight with 95% uncertainty interval.

Name of GBD health states	Definition	Disability Weight (95% UI)
Low Back Pain with Leg Pain		
Mild low back pain with leg pain	This person has mild back pain, which causes some difficulty dressing, standing, and lifting things.	0.020 (0.011–0.035)
Moderate low back pain with leg pain	This person has moderate back pain, which causes difficulty dressing, sitting, standing, walking, and lifting things.	0.054 (0.035–0.079)
Severe low back pain with leg pain	This person has severe back and leg pain, which causes difficulty dressing, sitting, standing, walking, and lifting things. The person sleeps poorly and feels worried.	0.325 0.219–0.446)
Most severe low back pain with leg pain	This person has constant back and leg pain, which causes difficulty dressing, sitting, standing, walking, and lifting things. The person sleeps poorly, is worried, and has lost some enjoyment in life.	0.384(0.256, 0.518)
Low Back Pain without Leg Pain		
Mild low back pain without leg pain	This person has mild back pain, which causes some difficulty dressing, standing, and lifting things.	0.020 (0.011–0.035)
Moderate low back pain without leg pain	This person has moderate back pain, which causes difficulty dressing, sitting, standing, walking, and lifting things.	0.054 (0.035–0.079)
Severe low back pain without leg pain	This person has severe back pain, which causes difficulty dressing, sitting, standing, walking, and lifting things. The person sleeps poorly and feels worried.	0.272(0.182, 0.373)
Most severe low back pain without leg pain	This person has constant back pain, which causes difficulty dressing, sitting, standing, walking, and lifting things. The person sleeps poorly, is worried, and has lost some enjoyment in life.	0.372(0.250, 0.0506)

Table S6. Results compared against WHO non-surgical guidelines.

Treatment	SMD	95% CI	Coverage % in LBP with leg pain (95% CI)	Coverage % in LBP without leg pain (95% CI)	WHO guidelines	Aligned with the results or not
Bed Rest	0.141	(-0.0103, 0.292)	Not surveyed	Not surveyed		
Education	-0.109	(-0.211, -0.00682)	Not surveyed	Not surveyed	Emphasize the importance of education and advice for patients, suggesting it should be a component of care.	Yes, our results indicate education provides benefits.

Epidural injection steroids	-0.178	(-0.299, -0.0570)	<0.1%	<0.1%	Generally not recommended as part of routine care.	Effective but concerns over the potential harms outweighing the minimal benefits offer in the short term
Non-opioid analgesics	-0.147	(-0.248, -0.0467)	33.2% (31.8%, 34.7%)	30.7% (30.0%, 31.4%)	Conditionally recommended, considered part of a broader treatment strategy, not a single intervention	Yes
Opioid analgesics	-0.209	(-0.324, -0.0952)	25.0% (23.7%, 26.4%)	12.4% (11.9%, 12.9%)	Generally, not recommended for routine use due to the risk of dependence and other ADEs.	Yes, the efficacy of opioid is still prominent.
Physical Intervention	-0.289	(-0.330, -0.248)	30.6% (29.2%, 32.0%)	20.6% (20.0%, 21.2%)	Strongly recommended	Yes
Psychological and Physical Interventions	-0.460	(-0.606, -0.309)	0.1% (0.0%, 0.2%)	<0.1%	Conditionally recommended	WHO guidelines emphasize integrating psychological and physical therapies.
Psychological	-0.255	(-0.401, -0.109)	1.0% (0.7%, 1.3%)	0.3% (0.3%, 0.4%)	Recommended as part of the management of CPLBP	Yes
Surgery	-0.366	(-0.525, -0.207)	10.1% (9.1%, 11.0%)	2.0% (1.8%, 2.2%)	Conditionally recommended when non-surgical treatments have been unsuccessful, also with clear indication.	Yes

Section 3: Estimation of LBP in current MEPS data

MEPS data

As for GBD 2021, Medical Expenditure Panel Survey(MEPS) was used to inform the severity distribution split for low back pain in GBD. MEPS is a national health survey, that began in 1996, and collected data from families and individuals, their medical providers, and employers across the USA[3]. MEPS collects information on participants' health system encounters, including self-reported underlying diseases, health service utilization, and healthcare expenditures. Panels are two-year-long in five rounds, with 30,000 to 35,000 individuals in each panel. MEP data started from 2000 to 2012 was used in the analysis because composite SF-12 scores began to be collected in 2000. We attempted to include more data; however, the occupational therapy, Physical intervention, and psychotherapy-related questions were removed from surveys after 2012.

Utilization of treatment

Individual utilization of pharmaceutical intervention is defined as the use of at least one medication in that intervention class in the therapeutic class. Utilization of surgery for treating LBP was assessed using MEPS hospital visits data including office-based medical provider visits, emergency room visits, outpatient department visits and hospital inpatient stays datasets.

Treatment utilization estimates were acquired from the MEPS household component event files which contain responses related to medical events through self-reported surveys[4]. The event files contain different types of clinical visit data: dental visits, office-based medical provider visits, inpatient hospitalization, emergency room visits, outpatient visits, and home health events. For the utilization estimation of healthcare interventions for LBP, the data was limited to the events that were associated with LBP. The classes in MEPS were aligned with the classes used in treatment effect estimation. The drug data was mapped to normalized drug names using the Multum Lexicon labels from the Cerner Multum. Inc. drug database. Individual utilization of surgical intervention was defined as if a patient received anesthesia and a surgical procedure due to LBP in at least one hospital/office visit.

Utilization of behavioral cognitive therapies and physical interventions were assessed using the MEPS office-based medical provider visits, outpatient visits, and home health files. These data contain information indicating if an individual received Physical intervention, occupational therapy, or psychotherapy during the visits. Moreover, the ICD-9 procedure codes associated with LBP were also retrieved as a complementary of the hospital visits data (first two characters of ICD-9 procedure=93 psychotherapy, =94 physical intervention). The utilization of combined therapies was defined as utilizing multiple treatments simultaneously for treating LBP. MEPS data spans from 2000 to 2012 and the panels after 2012 were not included because the treatment-related questions were removed from the survey.

The most frequently used treatment in LBP is non-opioid NSAID across all panels, followed by physical treatments. Surgery is often used in more severe LBP (Figure S6 Top). In the utilization of combined therapy, the most frequently used are cognitive behavioral therapies + physical interventions (Figure S6 Bottom).

Figure S6. Utilization plots using MEPS data by MEPS panel. Shaded areas represent a 95% confidence interval. Top: Individual treatment; Bottom: combined treatment.

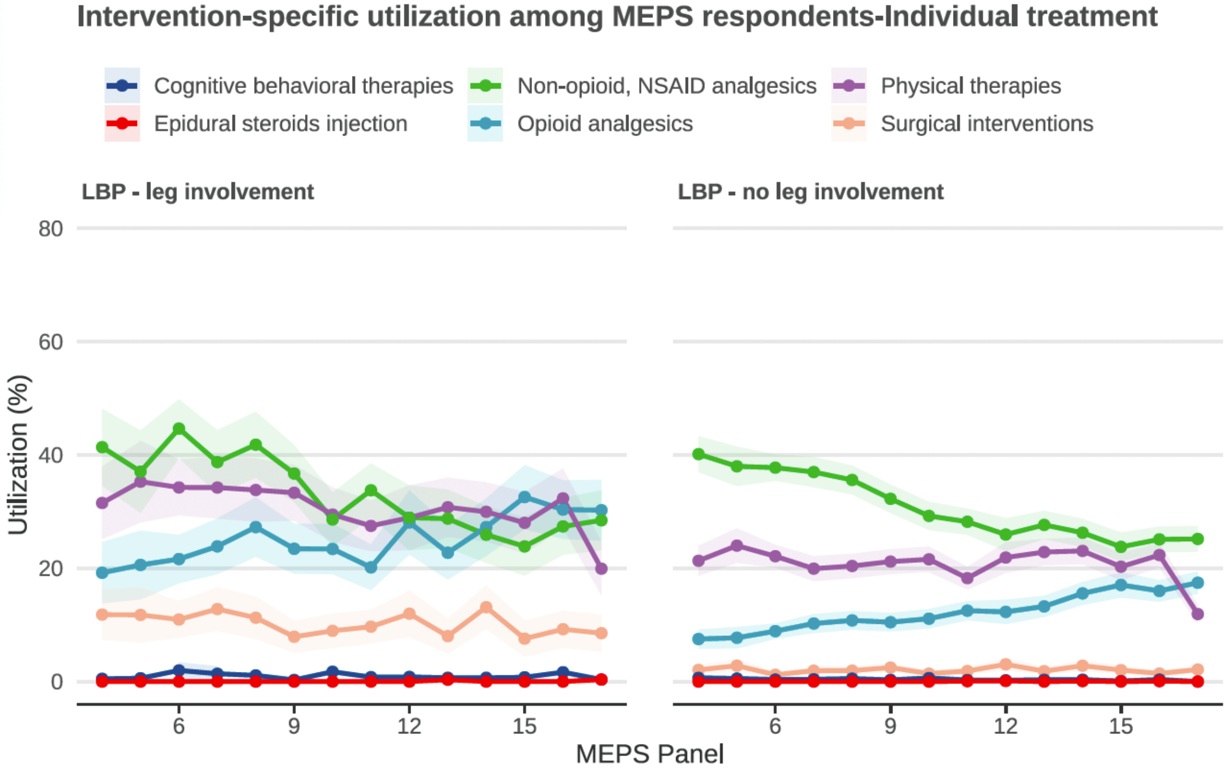

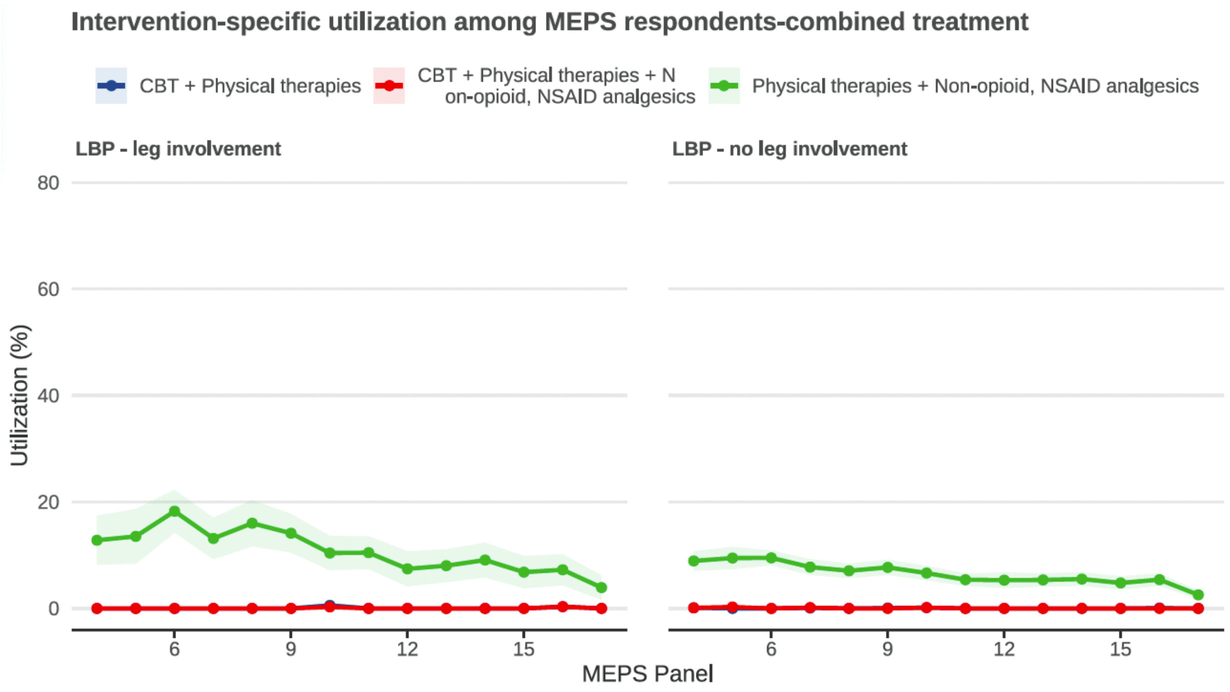

Section 4: Grading severity by healthcare access

The comorbidity corrected disability weights were mapped back to the SF-12 score and adjusted for obtaining the disability solely attributed to LBP. We defined the term as $SF12_{no\ treatment,i}$ and computed as following, where i is the individual MEPs respondent, and $SMD_{overall} * \sigma_{SF12}$ is the product of coverage adjusted treatment effect size and the standard deviation of the SF-12 for all participants.

$$SF12_{no\ treatment,i} = SF12_i + SMD_{overall} * \sigma_{SF12} \text{ (equation 1)}$$

For the full utilization optimal treatment scenario(FUOT), using $SF12_{no\ treatment,i}$ calculated from equation 2, $SF12_{Fuot,i}$ is calculated by multiplying the SF-12 SD with the FUOT intervention effect size and subtracting from the SF-12 score of the correspondent treatment to get the SF-12 for the FUOT scenario at patient-level (Equation 3).

$$SF12_{Fuot,i} = SF12_{no\ treatment,i} - SMD_{Fuot} * \sigma_{SF12} \text{(equation 2)}$$

Then both $SF12_{no\ treatment,i}$ and $SF12_{Fuot,i}$ was mapped back to disability weight and followed by binning them into severity specific-sequelae as asymptomatic, mild, moderate, severe, and most severe. The proportion and the disability weight of each severity-specific sequela for each scenario were calculated respectively (Figure S8).

The healthcare access quality index[5] (HAQI) was used as a proxy for treatment access due to the lack of data on treatment coverage for low back pain. The HAQI is informed by the mortality rate of 32 causes of death which should not, or rarely, occur in the presence of effective care. The index spans between 0 (worst) and 100 (best) representing the 1st and 99th percentile observed since 1990 and every location-year has a corresponding HAQI score.

$$AF = \frac{DW_{sequela-weighted,current\ treatment}}{DW_{sequela-weighted,no\ treatment}}$$

An adjustment factor(AF) to sequela-weighted disability weight was calculated using MEPS data as the ratio of disability weight with current treatment over disability weight with no treatment. The HAQI value is assumed to be 0 for the no-treatment scenario, where an individual does not have any access to LBP interventions and the adjustment factor equals 1 for the US in 2007, while the HAQI was 81.0 [79.5 to 82.5]. Assuming the association between HAQI and adjustment factor is linear, it was estimated that with 1 unit increase in HAQI would result in a

0.0037 [0.0037, 0.0037] unit increase in the adjustment factor (Figure S7). Using the 2020 HAQI, Iceland had the highest disease-specific accessibility 94.19[93.1, 95.1] globally, which is used in the full utilization optimal scenario. Central African Republic had the lowest HAQI 14.0[12.5, 15.5].

Figure S7. Adjustment factor to 2007 US Medical Expenditure Panel Survey sequela-weighted disability weight varying by healthcare access quality index

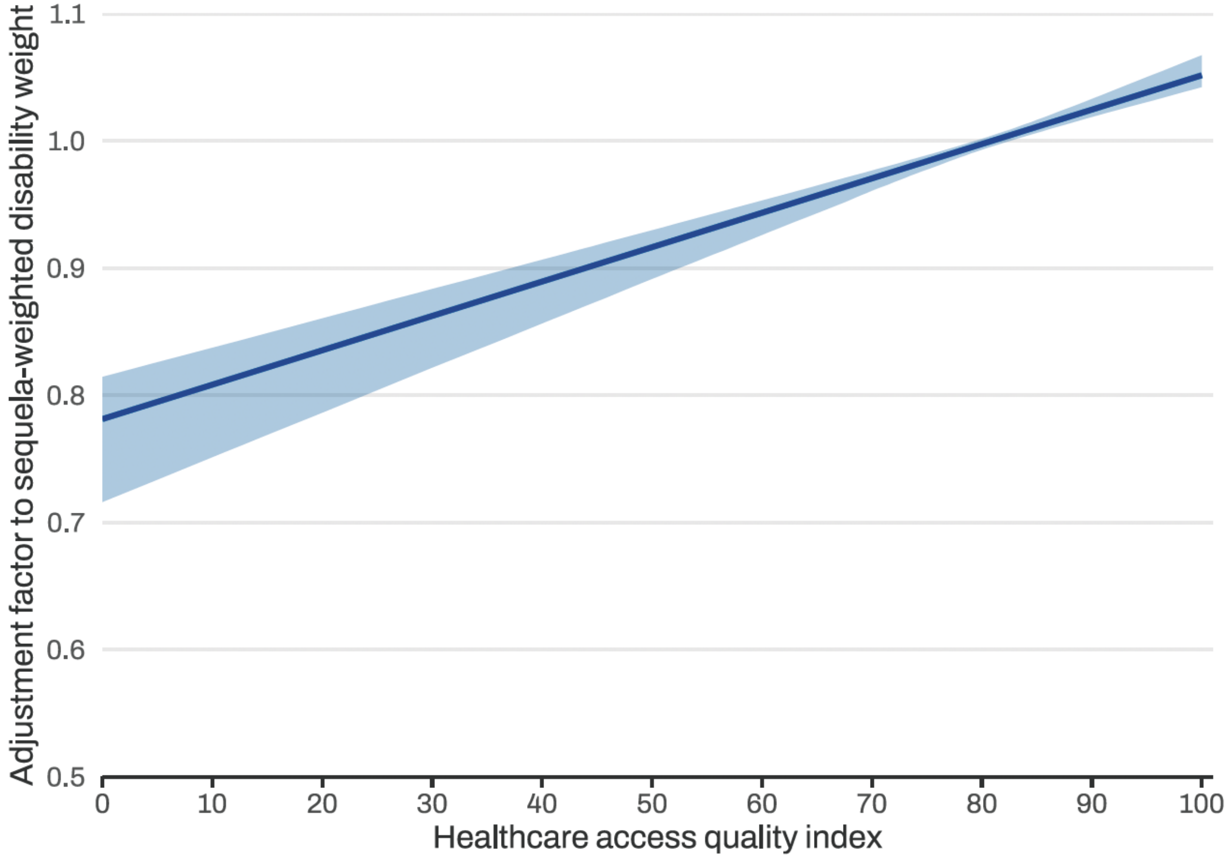

The Sequela-weighted disability weights were adjusted in the following way:

$$\frac{DW_{MEPS}}{AF_{HAQI}} = \sum_{s=mild}^{most\ severe}(DW_s^{MEPS} \times \frac{Proportion_s^{MEPS}}{AF_{HAQI}}) \text{ (equation 3)}$$

Where DW_{MEPS} represents the average disability weight from 2010 for low back pain, and adjusted by deviding the AF_{HAQI} which is the adjustment factor of HAQI. $Proportion_s^{MEPS}$ is the proportion of low back pain cases in severity s observed from USA MEPS data 2010, where s represents severity (mild, moderate, severe, most severe), and the HAQI was from the USA in 2010 (the year that corresponds to the midpoint of data collection years for MEPS).

Then the proportion for each health state s varying by HAQI using MEPS 2010 is calculated as:

$$Proportion_{s,HAQI} = \frac{Proportion_s^{MEPS}}{AF_{HAQI}} \text{ (equation 4)}$$

The proportion in the asymptomatic state was calculated as the residual proportion subtracting from 1:

$$Proportion_{asymp,HAQI} = 1 - \sum_{mild}^{most\ severe} Proportion_{s,HAQI} \text{(equation 5)}$$

Each experiment was repeated 1000 times to add uncertainty to the calculation. There are some draws where the proportion of asymptomatic cases is negative. To resolve this issue, we set the proportion of asymptomatic cases as 0 and repeated the similar method in equation 5. Instead of calculating the mild sequela residual proportion, we calculated the moderate sequela residual proportion. Followed by calculating the difference between the resulting disability weight when using mild sequela residual proportion as the residual proportion and the HAQI-specific sequela-weighted disability weight. Using this method, we ensure the sum of the proportion sequela is equal to one.

$$DW_{difference} = \sum_{moderate}^{the\ most\ severe}(DW_s \times Proportion_{s,HAQI}) + DW_{mild} \times \left(1 - \sum_{moderate}^{the\ most\ severe} Proportion_{s,HAQI}\right) - \frac{DW}{AF_{HAQI,2010}} \text{(equation 6)}$$

We re-iterate the process until all the severity proportions are non-negative. The correction for the most severe category is described as following as an example:

$$Proportion_{difference} = \frac{DW_{difference}}{DW_{severe} - DW_{most\ severe}} \text{(equation 7)}$$

$$Proportion_{most\ severe,HAQI} = \frac{Proportion_{most\ severe,MEPs}}{AF_{HAQI}} + Proportion_{difference} \text{(equation 8)}$$

The proportion in the severe sequela would be the new residual sequela proportion:

$$Proportion_{severe,HAQI} = 1 - Proportion_{most\ severe,HAQI} \text{(equation 9)}$$

Figure S8. MEPS disability weight distributions by the scenario in the MEPS sample for LBP with and without leg involvement. Density plots of three scenarios: 1) with no treatment 2) with current treatment 3) full utilization optimal treatment. Black lines represent the mean of the distribution.

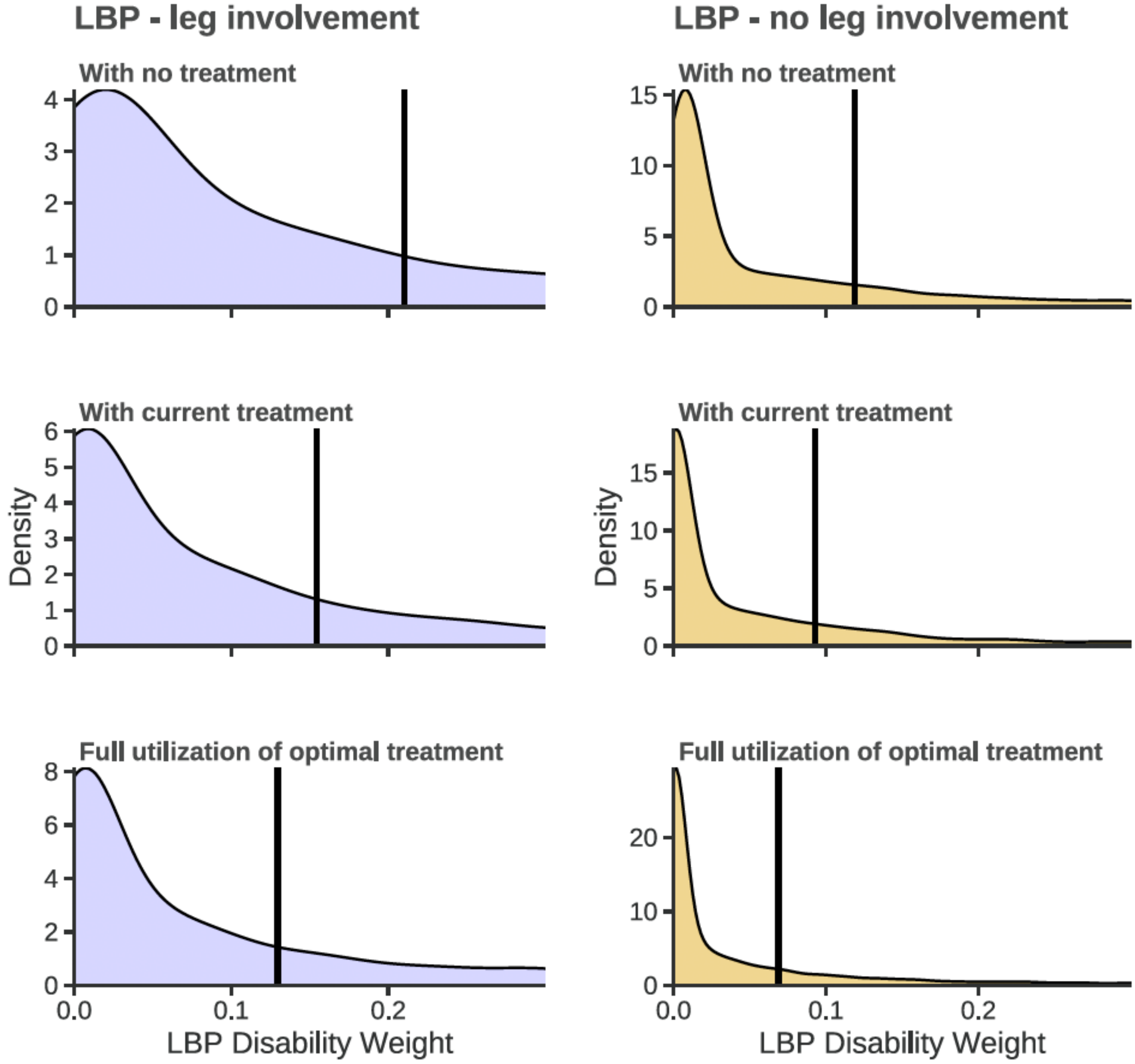

Figure S9. Distribution of disability weights among LBP cases for three scenarios (Upper Fig: LBP without leg pain; Lower Fig: LBP with leg pain. Dotted lines represent the cutoffs) in MEPS.

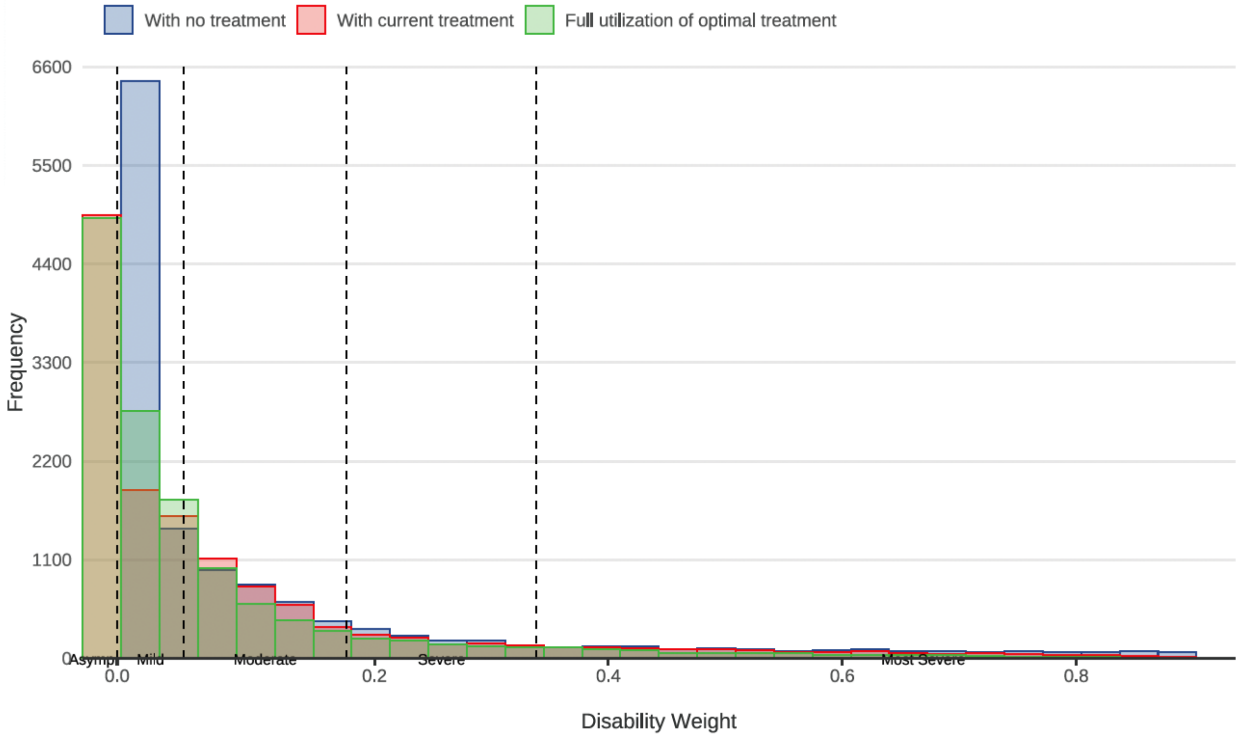

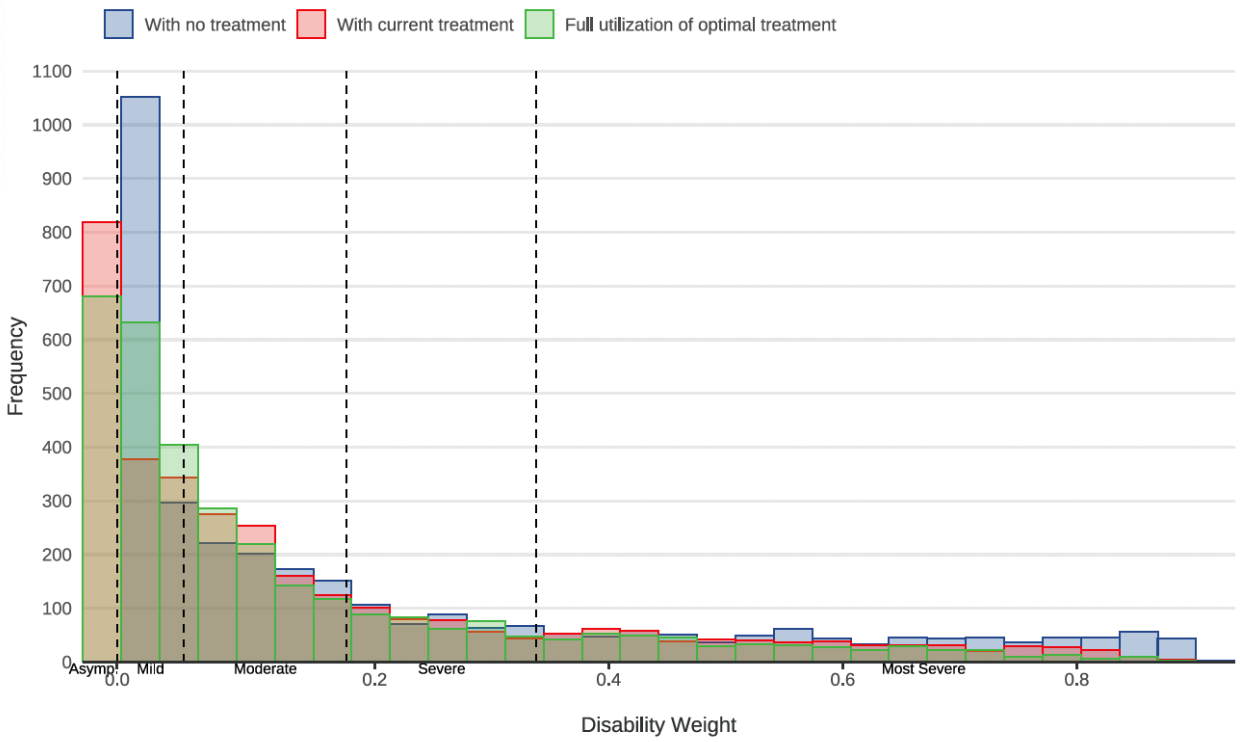

Table S6. Health Access and Quality index (HAQi) values and disability weights used in GBD 2021 and those estimated for the year 2020 with a gradient on access to treatment by country, region, and super-region

Location	HAQI	Original DW	Adjusted DW	DW % change
**Global**	55·6 (55·2–56·1)	0·096 (0·065–0·132)	0·102 (0·081–0·116)	12·1 (-15·5–42·0)
**Central Europe, eastern Europe, and central Asia**	67·1 (66·4–67·8)	0·096 (0·065–0·132)	0·099 (0·080–0·113)	9·3 (-17·4–37·6)
**Central Asia**	52·9 (52·1–53·9)	0·096 (0·065–0·132)	0·104 (0·083–0·119)	14·4 (-14·0–45·5)
Armenia	64·0 (62·0–66·0)	0·096 (0·065–0·132)	0·101 (0·069–0·140)	4·9 (3·9–6·7)
Azerbaijan	54·6 (53·5–56·5)	0·096 (0·065–0·132)	0·103 (0·071–0·145)	7·8 (6·3–10·4)
Georgia	58·0 (56·5–59·5)	0·096 (0·065–0·132)	0·102 (0·070–0·143)	6·8 (5·4–9·3)
Kazakhstan	60·8 (59·0–62·5)	0·096 (0·065–0·132)	0·102 (0·069–0·141)	5·9 (4·7–8·1)
Kyrgyzstan	55·1 (53·0–57·0)	0·096 (0·065–0·132)	0·103 (0·070–0·145)	7·7 (6·0–10·7)
Mongolia	47·3 (45·5–49·0)	0·096 (0·065–0·132)	0·106 (0·072–0·149)	10·2 (8·2–14·2)
Tajikistan	42·7 (40·5–44·5)	0·096 (0·065–0·132)	0·107 (0·073–0·151)	11·8 (9·4–16·3)
Turkmenistan	49·7 (47·5–51·5)	0·096 (0·065–0·132)	0·105 (0·071–0·148)	9·5 (7·5–13·6)
Uzbekistan	50·0 (48·5–52·0)	0·096 (0·065–0·132)	0·105 (0·071–0·147)	9·3 (7·6–13·3)
**Central Europe**	74·7 (73·8–75·4)	0·096 (0·065–0·132)	0·098 (0·079–0·111)	7·5 (-18·6–35·2)
Albania	70·8 (69·0–72·5)	0·096 (0·065–0·132)	0·099 (0·067–0·137)	2·9 (1·9–4·2)
Bosnia and Herzegovina	70·8 (69·0–72·0)	0·096 (0·065–0·132)	0·099 (0·067–0·137)	2·9 (2·1–4·0)
Bulgaria	66·5 (65·0–68·5)	0·096 (0·065–0·132)	0·100 (0·068–0·139)	4·1 (3·1–5·9)
Croatia	83·0 (81·0–84·5)	0·096 (0·065–0·132)	0·095 (0·065–0·131)	-0·6 (-1·1–0·0)
Czechia	82·2 (81·0–83·5)	0·096 (0·065–0·132)	0·096 (0·065–0·131)	-0·4 (-0·8–0·1)
Hungary	76·7 (75·5–78·0)	0·096 (0·065–0·132)	0·097 (0·066–0·134)	1·2 (0·5–1·8)
Montenegro	77·3 (75·5–78·5)	0·096 (0·065–0·132)	0·097 (0·066–0·133)	1·0 (0·4–1·8)
North Macedonia	69·5 (67·5–71·5)	0·096 (0·065–0·132)	0·099 (0·068–0·137)	3·2 (2·5–4·7)
Poland	74·6 (72·5–76·5)	0·096 (0·065–0·132)	0·098 (0·067–0·135)	1·8 (1·1–2·8)
Romania	70·9 (69·0–72·5)	0·096 (0·065–0·132)	0·099 (0·067–0·137)	2·9 (2·0–4·0)
Serbia	74·5 (72·0–76·5)	0·096 (0·065–0·132)	0·098 (0·067–0·135)	1·8 (1·0–3·0)
Slovakia	75·2 (73·5–77·0)	0·096 (0·065–0·132)	0·097 (0·067–0·135)	1·6 (1·0–2·4)
Slovenia	89·2 (88·0–90·5)	0·096 (0·065–0·132)	0·094 (0·064–0·129)	-2·2 (-3·0–-1·5)
**Eastern Europe**	69·5 (68·2–70·7)	0·096 (0·065–0·132)	0·099 (0·080–0·112)	9·2 (-17·5–37·4)
Belarus	74·0 (72·5–75·5)	0·096 (0·065–0·132)	0·098 (0·067–0·135)	1·9 (1·3–2·9)
Estonia	78·4 (76·5–80·5)	0·096 (0·065–0·132)	0·097 (0·066–0·133)	0·7 (-0·1–1·6)
Latvia	70·9 (69·0–72·5)	0·096 (0·065–0·132)	0·099 (0·068–0·136)	2·8 (2·1–3·9)
Lithuania	70·0 (68·0–72·0)	0·096 (0·065–0·132)	0·099 (0·068–0·137)	3·1 (2·3–4·4)
Moldova	64·7 (62·5–67·0)	0·096 (0·065–0·132)	0·100 (0·068–0·139)	4·7 (3·5–6·8)
Russia	70·6 (69·0–72·5)	0·096 (0·065–0·132)	0·099 (0·068–0·137)	2·9 (2·0–4·2)
Ukraine	64·7 (63·5–66·5)	0·096 (0·065–0·132)	0·100 (0·069–0·139)	4·7 (3·6–6·3)
**High income**	85·8 (85·2–86·3)	0·096 (0·065–0·132)	0·095 (0·076–0·107)	4·2 (-21·0–31·4)
**Australasia**	90·7 (89·7–91·7)	0·096 (0·065–0·132)	0·093 (0·076–0·105)	2·9 (-22·1–29·7)
Australia	91·4 (90·5–92·5)	0·096 (0·065–0·132)	0·093 (0·063–0·128)	-2·8 (-3·9–-2·2)
New Zealand	87·0 (85·5–88·5)	0·096 (0·065–0·132)	0·094 (0·064–0·130)	-1·7 (-2·4–-1·1)
**High-income Asia Pacific**	89·0 (88·1–90·0)	0·096 (0·065–0·132)	0·094 (0·076–0·106)	3·4 (-21·8–30·5)
Brunei	58·2 (56·0–60·0)	0·096 (0·065–0·132)	0·102 (0·070–0·143)	6·7 (5·3–8·9)
Japan	89·4 (88·0–91·0)	0·096 (0·065–0·132)	0·094 (0·064–0·129)	-2·3 (-3·3–-1·7)
South Korea	88·6 (87·5–90·0)	0·096 (0·065–0·132)	0·094 (0·064–0·129)	-2·1 (-2·9–-1·5)
Singapore	87·2 (86·0–88·5)	0·096 (0·065–0·132)	0·094 (0·064–0·130)	-1·7 (-2·5–-1·0)
**High-income North America**	83·6 (82·0–84·9)	0·096 (0·065–0·132)	0·095 (0·077–0·108)	4·9 (-20·5–32·2)
Canada	92·4 (91·0–93·5)	0·096 (0·065–0·132)	0·093 (0·063–0·128)	-3·1 (-4·2–-2·3)
Greenland	62·3 (61·0–64·0)	0·096 (0·065–0·132)	0·101 (0·069–0·141)	5·4 (4·2–7·6)
USA	82·6 (81·0–84·0)	0·096 (0·065–0·132)	0·095 (0·065–0·132)	-0·5 (-1·1–0·2)
**Southern Latin America**	64·6 (63·5–65·8)	0·096 (0·065–0·132)	0·100 (0·080–0·114)	10·6 (-16·5–39·6)
Argentina	61·0 (59·5–63·0)	0·096 (0·065–0·132)	0·101 (0·069–0·141)	5·8 (4·4–7·9)
Chile	73·1 (71·5–75·0)	0·096 (0·065–0·132)	0·098 (0·067–0·135)	2·2 (1·5–3·1)
Uruguay	65·9 (63·5–68·0)	0·096 (0·065–0·132)	0·100 (0·069–0·139)	4·3 (3·2–6·2)
**Western Europe**	89·2 (88·7–89·6)	0·096 (0·065–0·132)	0·094 (0·076–0·106)	3·3 (-21·8–30·2)
Andorra	91·3 (90·0–92·5)	0·096 (0·065–0·132)	0·093 (0·064–0·128)	-2·8 (-3·8–-2·1)
Austria	89·5 (88·0–90·5)	0·096 (0·065–0·132)	0·094 (0·064–0·129)	-2·3 (-3·4–-1·6)

Belgium	88·3 (86·5–90·0)	0·096 (0·065–0·132)	0·094 (0·064–0·129)	-2·0 (-2·8–-1·4)
Cyprus	88·5 (87·0–90·0)	0·096 (0·065–0·132)	0·094 (0·064–0·129)	-2·1 (-2·8–-1·4)
Denmark	87·2 (86·0–88·5)	0·096 (0·065–0·132)	0·094 (0·064–0·129)	-1·7 (-2·6–-1·1)
Finland	89·3 (88·0–90·5)	0·096 (0·065–0·132)	0·094 (0·064–0·128)	-2·3 (-3·2–-1·6)
France	89·8 (88·5–91·0)	0·096 (0·065–0·132)	0·094 (0·064–0·129)	-2·4 (-3·4–-1·7)
Germany	88·6 (87·0–90·0)	0·096 (0·065–0·132)	0·094 (0·064–0·129)	-2·1 (-2·9–-1·5)
Greece	85·7 (84·0–87·0)	0·096 (0·065–0·132)	0·095 (0·065–0·130)	-1·3 (-2·2–-0·8)
Iceland	94·1 (93·5–95·0)	0·096 (0·065–0·132)	0·092 (0·063–0·127)	-3·5 (-4·8–-2·7)
Ireland	92·0 (91·0–93·0)	0·096 (0·065–0·132)	0·093 (0·064–0·128)	-3·0 (-4·2–-2·2)
Israel	84·8 (83·5–86·0)	0·096 (0·065–0·132)	0·095 (0·065–0·130)	-1·1 (-1·8–-0·5)
Italy	91·6 (90·5–92·5)	0·096 (0·065–0·132)	0·093 (0·063–0·128)	-2·9 (-4·1–-2·1)
Luxembourg	89·4 (88·0–90·5)	0·096 (0·065–0·132)	0·094 (0·064–0·129)	-2·3 (-3·4–-1·6)
Malta	87·4 (86·0–89·0)	0·096 (0·065–0·132)	0·094 (0·064–0·129)	-1·8 (-2·5–-1·2)
Monaco	89·8 (88·5–91·0)	0·096 (0·065–0·132)	0·094 (0·064–0·129)	-2·4 (-3·3–-1·7)
Netherlands	92·4 (91·5–93·5)	0·096 (0·065–0·132)	0·093 (0·063–0·127)	-3·1 (-4·2–-2·4)
Norway	91·6 (90·5–93·0)	0·096 (0·065–0·132)	0·093 (0·064–0·128)	-2·9 (-4·0–-2·1)
Portugal	86·0 (84·5–87·5)	0·096 (0·065–0·132)	0·095 (0·065–0·130)	-1·4 (-2·1–-0·8)
San Marino	90·6 (89·5–91·5)	0·096 (0·065–0·132)	0·093 (0·064–0·128)	-2·6 (-3·5–-2·0)
Spain	91·6 (90·5–92·5)	0·096 (0·065–0·132)	0·093 (0·063–0·128)	-2·9 (-3·8–-2·1)
Sweden	91·7 (90·5–93·0)	0·096 (0·065–0·132)	0·093 (0·063–0·128)	-2·9 (-3·9–-2·2)
Switzerland	93·2 (92·0–94·0)	0·096 (0·065–0·132)	0·093 (0·063–0·127)	-3·3 (-4·4–-2·6)
UK	85·5 (84·0–87·0)	0·096 (0·065–0·132)	0·095 (0·064–0·130)	-1·2 (-1·9–-0·6)
**Latin America and Caribbean**	53·6 (52·8–54·5)	0·096 (0·065–0·132)	0·104 (0·083–0·119)	14·2 (-14·1–45·5)
**Andean Latin America**	55·3 (54·3–56·3)	0·096 (0·065–0·132)	0·103 (0·082–0·118)	13·8 (-14·3–44·6)
Bolivia	40·3 (39·0–42·5)	0·096 (0·065–0·132)	0·108 (0·073–0·153)	12·6 (10·2–17·0)
Ecuador	53·7 (52·0–55·5)	0·096 (0·065–0·132)	0·104 (0·071–0·145)	8·1 (6·5–11·4)
Peru	61·0 (59·0–63·0)	0·096 (0·065–0·132)	0·101 (0·069–0·142)	5·8 (4·6–8·1)
**Caribbean**	47·9 (47·1–48·6)	0·096 (0·065–0·132)	0·105 (0·083–0·121)	15·5 (-13·2–47·8)
Antigua and Barbuda	59·1 (57·5–61·5)	0·096 (0·065–0·132)	0·102 (0·070–0·143)	6·4 (5·1–8·9)
The Bahamas	52·8 (51·0–54·5)	0·096 (0·065–0·132)	0·104 (0·071–0·146)	8·4 (6·9–11·2)
Barbados	60·0 (58·0–62·0)	0·096 (0·065–0·132)	0·102 (0·070–0·142)	6·1 (4·9–8·4)
Belize	49·1 (47·0–51·0)	0·096 (0·065–0·132)	0·105 (0·072–0·148)	9·6 (7·8–13·5)
Bermuda	79·8 (78·0–81·0)	0·096 (0·065–0·132)	0·096 (0·066–0·132)	0·3 (-0·3–0·9)
Cuba	67·6 (66·0–69·5)	0·096 (0·065–0·132)	0·100 (0·068–0·138)	3·8 (2·9–5·4)
Dominica	45·8 (44·0–47·5)	0·096 (0·065–0·132)	0·106 (0·072–0·151)	10·8 (8·7–15·0)
Dominican Republic	46·2 (44·5–48·0)	0·096 (0·065–0·132)	0·106 (0·072–0·150)	10·6 (8·7–14·9)
Grenada	50·4 (48·5–52·0)	0·096 (0·065–0·132)	0·105 (0·071–0·147)	9·2 (7·2–13·0)
Guyana	36·7 (35·0–38·5)	0·096 (0·065–0·132)	0·109 (0·074–0·156)	13·9 (11·3–19·5)
Haiti	22·7 (21·0–24·5)	0·096 (0·065–0·132)	0·114 (0·077–0·164)	19·2 (15·4–28·1)
Jamaica	55·3 (53·5–57·5)	0·096 (0·065–0·132)	0·103 (0·070–0·144)	7·6 (6·2–10·3)
Puerto Rico	73·3 (72·0–75·5)	0·096 (0·065–0·132)	0·098 (0·067–0·136)	2·1 (1·4–3·2)
Saint Kitts and Nevis	51·9 (50·0–54·0)	0·096 (0·065–0·132)	0·104 (0·071–0·147)	8·7 (7·1–11·9)
Saint Lucia	53·0 (51·0–55·0)	0·096 (0·065–0·132)	0·104 (0·071–0·146)	8·4 (6·8–11·9)
Saint Vincent and the Grenadines	48·2 (46·0–50·0)	0·096 (0·065–0·132)	0·105 (0·072–0·148)	9·9 (8·0–13·8)
Suriname	42·8 (41·0–44·5)	0·096 (0·065–0·132)	0·107 (0·073–0·152)	11·8 (9·7–16·3)
Trinidad and Tobago	53·2 (51·0–55·0)	0·096 (0·065–0·132)	0·104 (0·071–0·146)	8·3 (6·5–11·7)
Virgin Islands	58·6 (56·5–60·5)	0·096 (0·065–0·132)	0·102 (0·070–0·143)	6·6 (5·1–9·1)
**Central Latin America**	54·5 (53·5–55·8)	0·096 (0·065–0·132)	0·103 (0·082–0·118)	13·9 (-14·2–44·6)
Colombia	62·2 (60·5–64·5)	0·096 (0·065–0·132)	0·101 (0·069–0·141)	5·5 (4·2–7·3)
Costa Rica	66·5 (64·5–68·0)	0·096 (0·065–0·132)	0·100 (0·068–0·139)	4·1 (3·3–5·7)
El Salvador	56·1 (55·0–57·5)	0·096 (0·065–0·132)	0·103 (0·071–0·144)	7·3 (5·8–10·5)
Guatemala	43·0 (40·5–45·0)	0·096 (0·065–0·132)	0·107 (0·073–0·151)	11·7 (9·5–16·1)
Honduras	39·1 (37·5–41·0)	0·096 (0·065–0·132)	0·108 (0·074–0·153)	13·1 (10·6–18·3)
Mexico	53·6 (52·0–56·0)	0·096 (0·065–0·132)	0·104 (0·071–0·145)	8·1 (6·5–11·0)
Nicaragua	53·4 (51·5–55·0)	0·096 (0·065–0·132)	0·104 (0·071–0·146)	8·2 (6·6–11·2)
Panama	61·2 (59·5–63·0)	0·096 (0·065–0·132)	0·101 (0·069–0·141)	5·8 (4·4–8·3)
Venezuela	54·2 (52·5–56·5)	0·096 (0·065–0·132)	0·104 (0·071–0·145)	8·0 (6·4–10·7)
**Tropical Latin America**	53·3 (52·0–55·4)	0·096 (0·065–0·132)	0·104 (0·083–0·120)	14·4 (-14·0–45·5)

Brazil	53·4 (52·0–55·5)	0·096 (0·065–0·132)	0·104 (0·071–0·146)	8·2 (6·6–11·6)
Paraguay	52·9 (51·0–55·0)	0·096 (0·065–0·132)	0·104 (0·071–0·145)	8·4 (6·7–11·5)
**North Africa and Middle East**	57·3 (56·8–57·7)	0·096 (0·065–0·132)	0·102 (0·082–0·117)	12·7 (-15·0–43·3)
Afghanistan	28·4 (26·5–30·5)	0·096 (0·065–0·132)	0·112 (0·076–0·161)	17·0 (13·7–23·8)
Algeria	60·2 (59·0–62·0)	0·096 (0·065–0·132)	0·102 (0·070–0·142)	6·0 (4·7–8·1)
Bahrain	69·9 (68·0–71·5)	0·096 (0·065–0·132)	0·099 (0·068–0·137)	3·1 (2·2–4·5)
Egypt	53·1 (51·5–55·0)	0·096 (0·065–0·132)	0·104 (0·071–0·145)	8·3 (6·7–11·2)
Iran	65·9 (64·0–67·5)	0·096 (0·065–0·132)	0·100 (0·068–0·139)	4·3 (3·4–5·9)
Iraq	59·5 (58·0–61·0)	0·096 (0·065–0·132)	0·102 (0·069–0·143)	6·3 (4·9–8·6)
Jordan	66·9 (65·0–68·5)	0·096 (0·065–0·132)	0·100 (0·068–0·138)	4·0 (3·1–5·2)
Kuwait	78·5 (76·5–80·0)	0·096 (0·065–0·132)	0·097 (0·066–0·133)	0·7 (0·1–1·3)
Lebanon	71·7 (69·5–73·5)	0·096 (0·065–0·132)	0·098 (0·067–0·136)	2·6 (1·7–3·8)
Libya	59·7 (58·0–61·5)	0·096 (0·065–0·132)	0·102 (0·069–0·142)	6·2 (4·9–8·6)
Morocco	50·0 (48·0–51·5)	0·096 (0·065–0·132)	0·105 (0·071–0·147)	9·3 (7·7–12·9)
Oman	69·0 (67·5–71·0)	0·096 (0·065–0·132)	0·099 (0·068–0·137)	3·4 (2·5–4·6)
Palestine	59·6 (57·5–61·0)	0·096 (0·065–0·132)	0·102 (0·070–0·143)	6·2 (5·0–8·5)
Qatar	77·6 (76·0–79·0)	0·096 (0·065–0·132)	0·097 (0·066–0·133)	0·9 (0·3–1·6)
Saudi Arabia	65·9 (64·0–67·5)	0·096 (0·065–0·132)	0·100 (0·068–0·139)	4·3 (3·3–5·7)
Sudan	44·5 (43·0–46·0)	0·096 (0·065–0·132)	0·107 (0·073–0·150)	11·2 (9·0–15·6)
Syria	62·4 (60·5–64·5)	0·096 (0·065–0·132)	0·101 (0·069–0·141)	5·4 (4·2–7·4)
Tunisia	66·2 (64·5–68·0)	0·096 (0·065–0·132)	0·100 (0·068–0·139)	4·2 (3·3–5·6)
Türkiye	67·0 (65·0–69·0)	0·096 (0·065–0·132)	0·100 (0·068–0·139)	4·0 (2·9–5·8)
United Arab Emirates	60·0 (58·5–62·0)	0·096 (0·065–0·132)	0·102 (0·070–0·142)	6·1 (5·1–8·3)
Yemen	39·2 (37·0–41·0)	0·096 (0·065–0·132)	0·108 (0·074–0·154)	13·1 (10·7–18·6)
**South Asia**	39·4 (37·9–41·1)	0·096 (0·065–0·132)	0·108 (0·086–0·126)	19·3 (-10·5–53·3)
Bangladesh	45·5 (44·0–47·5)	0·096 (0·065–0·132)	0·106 (0·072–0·150)	10·9 (8·8–14·6)
Bhutan	43·4 (41·0–45·0)	0·096 (0·065–0·132)	0·107 (0·073–0·151)	11·6 (9·3–16·0)
India	39·8 (38·0–42·0)	0·096 (0·065–0·132)	0·108 (0·074–0·153)	12·9 (10·6–18·1)
Nepal	39·9 (38·0–41·5)	0·096 (0·065–0·132)	0·108 (0·073–0·153)	12·8 (10·4–17·3)
Pakistan	32·9 (31·0–35·0)	0·096 (0·065–0·132)	0·111 (0·075–0·157)	15·3 (12·3–21·5)
**Southeast Asia, east Asia, and Oceania**	64·4 (63·4–65·3)	0·096 (0·065–0·132)	0·100 (0·080–0·114)	10·5 (-16·4–39·5)
**East Asia**	72·9 (71·3–74·2)	0·096 (0·065–0·132)	0·098 (0·079–0·111)	8·1 (-18·0–36·0)
China	73·2 (71·5–74·5)	0·096 (0·065–0·132)	0·098 (0·067–0·135)	2·2 (1·6–3·1)
North Korea	51·8 (50·0–54·0)	0·096 (0·065–0·132)	0·104 (0·071–0·146)	8·8 (7·2–12·4)
Taiwan (province of China)	80·0 (78·5–81·5)	0·096 (0·065–0·132)	0·096 (0·065–0·133)	0·2 (-0·3–0·9)
**Oceania**	31·6 (29·6–33·2)	0·096 (0·065–0·132)	0·111 (0·087–0·130)	22·2 (-8·9–58·5)
American Samoa	46·3 (44·5–49·0)	0·096 (0·065–0·132)	0·106 (0·072–0·150)	10·6 (8·5–14·8)
Cook Islands	65·4 (63·5–67·0)	0·096 (0·065–0·132)	0·100 (0·069–0·139)	4·5 (3·6–6·2)
Fiji	38·9 (37·0–40·5)	0·096 (0·065–0·132)	0·109 (0·074–0·154)	13·1 (10·6–18·6)
Guam	58·2 (56·5–60·5)	0·096 (0·065–0·132)	0·102 (0·070–0·143)	6·7 (5·2–9·3)
Kiribati	23·4 (22·0–24·5)	0·096 (0·065–0·132)	0·114 (0·077–0·164)	18·9 (15·4–27·1)
Marshall Islands	31·5 (29·5–33·5)	0·096 (0·065–0·132)	0·111 (0·075–0·159)	15·8 (13·0–22·4)
Federated States of Micronesia	35·5 (34·0–37·5)	0·096 (0·065–0·132)	0·110 (0·074–0·156)	14·4 (11·6–20·1)
Nauru	36·4 (34·5–38·5)	0·096 (0·065–0·132)	0·109 (0·074–0·155)	14·1 (11·3–19·9)
Niue	49·7 (48·0–51·0)	0·096 (0·065–0·132)	0·105 (0·071–0·147)	9·4 (7·8–12·8)
Northern Mariana Islands	56·9 (55·0–58·5)	0·096 (0·065–0·132)	0·103 (0·070–0·143)	7·1 (5·7–9·4)
Palau	49·6 (47·5–51·5)	0·096 (0·065–0·132)	0·105 (0·071–0·148)	9·5 (7·7–13·0)
Papua New Guinea	30·1 (27·5–32·0)	0·096 (0·065–0·132)	0·112 (0·076–0·161)	16·4 (13·2–23·2)
Samoa	44·4 (42·5–46·0)	0·096 (0·065–0·132)	0·107 (0·073–0·150)	11·2 (9·1–15·5)
Solomon Islands	29·6 (27·5–31·5)	0·096 (0·065–0·132)	0·112 (0·076–0·160)	16·6 (13·4–23·5)
Tokelau	47·0 (45·0–48·5)	0·096 (0·065–0·132)	0·106 (0·072–0·149)	10·3 (8·1–14·4)
Tonga	46·7 (45·0–49·0)	0·096 (0·065–0·132)	0·106 (0·072–0·149)	10·4 (8·5–14·4)
Tuvalu	38·1 (36·5–39·5)	0·096 (0·065–0·132)	0·109 (0·074–0·154)	13·4 (10·7–18·3)
Vanuatu	30·5 (28·5–32·5)	0·096 (0·065–0·132)	0·112 (0·075–0·160)	16·2 (13·1–23·2)
**Southeast Asia**	47·1 (46·0–47·9)	0·096 (0·065–0·132)	0·106 (0·084–0·122)	16·5 (-12·6–49·5)
Cambodia	38·7 (37·0–40·5)	0·096 (0·065–0·132)	0·109 (0·074–0·154)	13·2 (10·5–18·9)
Indonesia	41·2 (38·5–43·5)	0·096 (0·065–0·132)	0·108 (0·073–0·153)	12·4 (10·0–17·3)
Laos	33·0 (31·0–35·0)	0·096 (0·065–0·132)	0·111 (0·075–0·158)	15·3 (12·3–21·3)

Malaysia	56·9 (55·0–59·0)	0·096 (0·065–0·132)	0·103 (0·070–0·143)	7·1 (5·8–9·6)
Maldives	62·2 (60·5–64·0)	0·096 (0·065–0·132)	0·101 (0·069–0·141)	5·5 (4·4–7·6)
Mauritius	57·6 (55·5–59·5)	0·096 (0·065–0·132)	0·103 (0·070–0·143)	6·9 (5·6–9·5)
Myanmar	38·4 (36·5–40·0)	0·096 (0·065–0·132)	0·109 (0·074–0·155)	13·3 (10·8–18·8)
Philippines	41·3 (39·0–43·0)	0·096 (0·065–0·132)	0·108 (0·073–0·153)	12·3 (10·0–17·5)
Seychelles	54·5 (52·5–56·5)	0·096 (0·065–0·132)	0·103 (0·071–0·144)	7·9 (6·3–10·8)
Sri Lanka	63·1 (61·0–65·0)	0·096 (0·065–0·132)	0·101 (0·069–0·141)	5·2 (3·9–7·2)
Thailand	64·9 (63·5–66·5)	0·096 (0·065–0·132)	0·100 (0·069–0·140)	4·6 (3·4–6·4)
Timor-Leste	35·1 (33·0–36·5)	0·096 (0·065–0·132)	0·110 (0·075–0·156)	14·5 (11·9–20·3)
Viet Nam	58·7 (57·0–61·0)	0·096 (0·065–0·132)	0·102 (0·070–0·143)	6·5 (5·2–8·9)
**Sub-Saharan Africa**	31·3 (30·7–31·7)	0·096 (0·065–0·132)	0·111 (0·088–0·130)	22·5 (-8·6–59·1)
**Central sub-Saharan Africa**	28·3 (27·2–29·2)	0·096 (0·065–0·132)	0·112 (0·088–0·132)	23·7 (-8·0–61·2)
Angola	29·8 (28·0–32·0)	0·096 (0·065–0·132)	0·112 (0·076–0·159)	16·5 (13·4–23·2)
Central African Republic	14·1 (13·0–15·5)	0·096 (0·065–0·132)	0·118 (0·079–0·171)	22·7 (18·2–33·1)
Congo (Brazzaville)	33·1 (31·5–35·0)	0·096 (0·065–0·132)	0·111 (0·075–0·157)	15·3 (12·4–21·3)
DR Congo	27·9 (26·0–29·0)	0·096 (0·065–0·132)	0·112 (0·076–0·161)	17·2 (13·7–24·7)
Equatorial Guinea	42·8 (41·0–44·5)	0·096 (0·065–0·132)	0·107 (0·073–0·151)	11·8 (9·7–16·3)
Gabon	39·6 (38·0–41·5)	0·096 (0·065–0·132)	0·108 (0·074–0·154)	12·9 (10·4–18·1)
**Eastern sub-Saharan Africa**	29·9 (29·3–30·5)	0·096 (0·065–0·132)	0·112 (0·088–0·131)	23·0 (-8·4–60·0)
Burundi	24·0 (22·5–25·5)	0·096 (0·065–0·132)	0·114 (0·077–0·164)	18·7 (15·1–26·5)
Comoros	31·1 (29·0–33·5)	0·096 (0·065–0·132)	0·111 (0·076–0·158)	16·0 (13·0–22·4)
Djibouti	32·8 (31·0–35·0)	0·096 (0·065–0·132)	0·111 (0·075–0·158)	15·4 (12·1–21·5)
Eritrea	24·5 (22·5–26·0)	0·096 (0·065–0·132)	0·114 (0·077–0·163)	18·5 (15·1–26·1)
Ethiopia	31·5 (30·0–34·0)	0·096 (0·065–0·132)	0·111 (0·076–0·159)	15·8 (12·7–22·4)
Kenya	32·9 (31·0–34·5)	0·096 (0·065–0·132)	0·111 (0·075–0·158)	15·3 (12·1–21·3)
Madagascar	27·8 (26·0–29·0)	0·096 (0·065–0·132)	0·112 (0·076–0·161)	17·2 (13·7–24·4)
Malawi	29·6 (28·0–31·5)	0·096 (0·065–0·132)	0·112 (0·076–0·159)	16·5 (13·8–23·0)
Mozambique	24·7 (23·0–26·5)	0·096 (0·065–0·132)	0·114 (0·077–0·164)	18·4 (14·6–26·4)
Rwanda	31·7 (30·0–33·5)	0·096 (0·065–0·132)	0·111 (0·075–0·159)	15·8 (12·8–22·2)
Somalia	16·6 (14·5–18·0)	0·096 (0·065–0·132)	0·117 (0·079–0·169)	21·7 (17·3–31·0)
South Sudan	28·3 (26·5–30·0)	0·096 (0·065–0·132)	0·112 (0·076–0·160)	17·1 (13·8–24·1)
Uganda	32·2 (30·5–34·0)	0·096 (0·065–0·132)	0·111 (0·075–0·158)	15·6 (12·7–21·3)
Tanzania	32·7 (31·0–34·5)	0·096 (0·065–0·132)	0·111 (0·075–0·157)	15·4 (12·5–22·0)
Zambia	31·3 (29·0–33·0)	0·096 (0·065–0·132)	0·111 (0·076–0·158)	15·9 (13·0–22·2)
**Southern sub-Saharan Africa**	40·4 (39·1–42·0)	0·096 (0·065–0·132)	0·108 (0·085–0·126)	18·9 (-11·0–52·8)
Botswana	37·8 (36·0–39·5)	0·096 (0·065–0·132)	0·109 (0·074–0·154)	13·5 (11·0–19·1)
Eswatini	32·9 (31·5–34·5)	0·096 (0·065–0·132)	0·111 (0·075–0·158)	15·3 (12·4–21·4)
Lesotho	25·6 (24·0–27·5)	0·096 (0·065–0·132)	0·113 (0·076–0·163)	18·1 (14·6–25·3)
Namibia	39·8 (38·5–42·0)	0·096 (0·065–0·132)	0·108 (0·074–0·153)	12·8 (10·4–17·6)
South Africa	44·5 (42·5–46·5)	0·096 (0·065–0·132)	0·107 (0·073–0·151)	11·2 (8·9–16·0)
Zimbabwe	28·2 (26·5–30·0)	0·096 (0·065–0·132)	0·112 (0·076–0·160)	17·1 (13·7–24·1)
**Western sub-Saharan Africa**	31·8 (30·7–32·7)	0·096 (0·065–0·132)	0·111 (0·088–0·130)	22·3 (-8·5–58·7)
Benin	30·9 (29·0–33·0)	0·096 (0·065–0·132)	0·111 (0·076–0·160)	16·1 (13·1–22·9)
Burkina Faso	28·1 (26·5–29·5)	0·096 (0·065–0·132)	0·112 (0·076–0·161)	17·1 (13·9–23·8)
Cabo Verde	51·3 (49·5–53·0)	0·096 (0·065–0·132)	0·104 (0·071–0·147)	8·9 (7·3–12·3)
Cameroon	33·1 (31·5–34·5)	0·096 (0·065–0·132)	0·111 (0·075–0·158)	15·3 (12·4–21·6)
Chad	23·4 (22·0–25·0)	0·096 (0·065–0·132)	0·114 (0·077–0·164)	18·9 (15·2–26·8)
Côte d'Ivoire	33·7 (32·0–36·0)	0·096 (0·065–0·132)	0·110 (0·075–0·157)	15·0 (12·2–20·6)
The Gambia	34·2 (32·5–35·5)	0·096 (0·065–0·132)	0·110 (0·075–0·157)	14·8 (12·1–20·8)
Ghana	36·3 (34·0–38·0)	0·096 (0·065–0·132)	0·109 (0·074–0·156)	14·1 (11·4–19·7)
Guinea	26·0 (24·0–28·0)	0·096 (0·065–0·132)	0·113 (0·076–0·161)	17·9 (14·5–25·2)
Guinea-Bissau	23·3 (21·0–25·0)	0·096 (0·065–0·132)	0·114 (0·077–0·164)	19·0 (15·5–26·5)
Liberia	34·5 (33·0–36·5)	0·096 (0·065–0·132)	0·110 (0·075–0·157)	14·7 (12·1–21·0)
Mali	29·2 (26·5–31·0)	0·096 (0·065–0·132)	0·112 (0·076–0·159)	16·7 (13·4–23·5)
Mauritania	41·6 (40·0–43·5)	0·096 (0·065–0·132)	0·108 (0·073–0·152)	12·2 (9·8–17·0)
Niger	25·6 (24·0–27·5)	0·096 (0·065–0·132)	0·113 (0·077–0·163)	18·1 (14·7–25·9)
Nigeria	32·6 (30·5–34·5)	0·096 (0·065–0·132)	0·111 (0·075–0·158)	15·4 (12·7–21·8)
São Tomé and Príncipe	42·1 (40·5–44·0)	0·096 (0·065–0·132)	0·107 (0·073–0·152)	12·0 (9·8–16·3)

Senegal	33·8 (32·0–36·0)	0·096 (0·065–0·132)	0·110 (0·075–0·157)	15·0 (12·0–20·8)
Sierra Leone	30·2 (29·0–32·0)	0·096 (0·065–0·132)	0·112 (0·076–0·159)	16·3 (13·3–23·0)
Togo	32·9 (31·0–34·5)	0·096 (0·065–0·132)	0·111 (0·075–0·158)	15·3 (12·5–22·3)

Table S7: Predicted severity proportions for low back pain for 2020 by country, region and super-region using the data with current treatment

Location	Asymptomatic	Mild	Moderate	Severe	Most_severe
Global	27·5 (25·5–28·5)	19·6 (14·2–25·6)	28·4 (22·4–33·4)	10·2 (8·6–12·1)	14·3 (9·1–21·1)
Central Europe, eastern Europe, and central Asia	30·1 (29·1–30·7)	18·9 (13·8–24·8)	27·4 (21·6–31·8)	9·8 (8·3–11·7)	13·8 (8·6–20·5)
Central Asia	27·2 (25·0–28·2)	19·7 (14·3–25·7)	28·6 (22·5–33·5)	10·2 (8·7–12·2)	14·3 (9·2–21·2)
Armenia	29·5 (22·0–36·8)	19·0 (13·9–24·9)	27·7 (21·7–32·1)	9·9 (8·4–11·9)	13·9 (4·2–25·2)
Azerbaijan	27·6 (19·4–35·2)	19·6 (14·2–25·6)	28·4 (22·5–33·2)	10·2 (8·6–12·1)	14·3 (4·3–25·8)
Georgia	28·3 (20·5–35·8)	19·4 (14·1–25·4)	28·1 (22·2–33·0)	10·1 (8·5–12·0)	14·1 (4·2–25·6)
Kazakhstan	28·9 (21·2–36·2)	19·2 (14·0–25·1)	27·9 (21·9–32·5)	10·0 (8·5–12·0)	14·0 (4·2–25·3)
Kyrgyzstan	27·7 (19·6–35·3)	19·5 (14·2–25·6)	28·4 (22·5–33·3)	10·2 (8·6–12·1)	14·2 (4·2–25·8)
Mongolia	25·9 (17·3–34·0)	20·0 (14·5–26·1)	29·1 (22·8–34·2)	10·4 (8·8–12·4)	14·6 (4·3–26·4)
Tajikistan	24·9 (16·1–33·2)	20·3 (14·6–26·6)	29·5 (23·2–34·7)	10·6 (8·9–12·6)	14·8 (4·4–26·7)
Turkmenistan	26·5 (18·0–34·3)	19·9 (14·4–25·9)	28·9 (22·7–33·8)	10·3 (8·8–12·3)	14·5 (4·3–26·2)
Uzbekistan	26·6 (18·1–34·5)	19·8 (14·4–26·0)	28·8 (22·7–33·8)	10·3 (8·8–12·3)	14·5 (4·3–26·2)
Central Europe	31·6 (31·0–32·1)	18·4 (13·5–24·2)	26·8 (21·1–30·9)	9·6 (8·1–11·5)	13·5 (8·4–20·1)
Albania	30·9 (23·9–37·9)	18·6 (13·7–24·5)	27·1 (21·3–31·3)	9·7 (8·2–11·7)	13·6 (4·1–24·8)
Bosnia and Herzegovina	30·9 (23·9–38·1)	18·6 (13·6–24·4)	27·1 (21·3–31·3)	9·7 (8·2–11·6)	13·6 (4·1–24·7)
Bulgaria	30·0 (22·8–37·4)	18·9 (13·8–24·7)	27·5 (21·6–31·8)	9·8 (8·3–11·8)	13·8 (4·1–25·0)
Croatia	33·2 (26·2–39·8)	18·0 (13·2–23·6)	26·2 (20·6–30·3)	9·4 (8·0–11·3)	13·2 (4·0–24·0)
Czechia	33·1 (26·1–39·8)	18·1 (13·3–23·6)	26·3 (20·7–30·3)	9·4 (8·0–11·3)	13·2 (4·0–24·0)
Hungary	32·0 (25·1–39·1)	18·3 (13·5–24·1)	26·7 (21·0–30·7)	9·6 (8·1–11·5)	13·4 (4·0–24·3)
Montenegro	32·2 (25·2–39·0)	18·3 (13·4–24·0)	26·6 (20·9–30·8)	9·5 (8·1–11·5)	13·4 (4·0–24·3)
North Macedonia	30·7 (23·6–37·8)	18·7 (13·8–24·5)	27·2 (21·5–31·6)	9·8 (8·3–11·7)	13·7 (4·1–24·8)
Poland	31·6 (24·7–38·6)	18·4 (13·5–24·3)	26·8 (21·1–30·9)	9·6 (8·2–11·5)	13·5 (4·0–24·5)
Romania	30·9 (23·8–38·0)	18·6 (13·7–24·4)	27·1 (21·3–31·4)	9·7 (8·2–11·7)	13·6 (4·1–24·7)
Serbia	31·6 (24·6–38·5)	18·5 (13·5–24·1)	26·8 (21·2–31·1)	9·6 (8·1–11·5)	13·5 (4·0–24·6)
Slovakia	31·8 (24·9–38·7)	18·4 (13·5–24·1)	26·8 (21·1–30·9)	9·6 (8·1–11·5)	13·4 (4·0–24·5)
Slovenia	34·3 (27·5–41·0)	17·7 (13·1–23·2)	25·8 (20·3–29·6)	9·2 (7·8–11·2)	12·9 (3·9–23·6)
Eastern Europe	30·6 (29·8–31·2)	18·7 (13·7–24·6)	27·2 (21·4–31·5)	9·8 (8·3–11·7)	13·7 (8·6–20·3)
Belarus	31·5 (24·4–38·5)	18·5 (13·5–24·3)	26·9 (21·2–31·1)	9·6 (8·2–11·5)	13·5 (4·1–24·5)
Estonia	32·4 (25·3–39·2)	18·2 (13·4–24·0)	26·5 (20·9–30·6)	9·5 (8·1–11·4)	13·3 (4·0–24·3)
Latvia	30·9 (23·7–38·0)	18·6 (13·7–24·4)	27·1 (21·3–31·3)	9·7 (8·2–11·7)	13·6 (4·1–24·8)
Lithuania	30·7 (23·6–37·8)	18·7 (13·7–24·5)	27·2 (21·3–31·5)	9·7 (8·2–11·7)	13·6 (4·1–24·6)
Moldova	29·7 (22·3–37·0)	19·0 (13·9–24·8)	27·6 (21·7–31·9)	9·9 (8·4–11·8)	13·8 (4·1–25·1)
Russia	30·9 (23·6–37·9)	18·7 (13·7–24·5)	27·1 (21·3–31·4)	9·7 (8·2–11·6)	13·6 (4·1–24·7)
Ukraine	29·7 (22·3–37·0)	19·0 (13·8–24·9)	27·6 (21·7–31·9)	9·9 (8·4–11·8)	13·8 (4·1–25·1)
High income	33·7 (33·3–34·1)	17·9 (13·2–23·4)	26·0 (20·5–30·0)	9·3 (7·9–11·2)	13·1 (8·0–19·6)
Australasia	34·6 (34·1–35·1)	17·6 (13·0–23·1)	25·7 (20·2–29·5)	9·2 (7·8–11·1)	12·9 (7·8–19·4)
Australia	34·7 (27·8–41·2)	17·6 (13·0–23·1)	25·6 (20·2–29·4)	9·2 (7·8–11·1)	12·9 (3·9–23·4)
New Zealand	33·9 (27·0–40·5)	17·8 (13·2–23·4)	25·9 (20·4–29·9)	9·3 (7·9–11·2)	13·0 (3·9–23·7)
High-income Asia Pacific	34·3 (33·8–34·9)	17·7 (13·1–23·3)	25·8 (20·3–29·6)	9·2 (7·8–11·2)	12·9 (7·9–19·4)
Brunei	28·3 (20·7–35·8)	19·3 (14·1–25·3)	28·1 (22·3–32·8)	10·1 (8·6–12·0)	14·1 (4·2–25·5)
Japan	34·4 (27·5–41·0)	17·7 (13·1–23·3)	25·8 (20·3–29·6)	9·2 (7·8–11·2)	12·9 (3·9–23·6)
South Korea	34·2 (27·4–40·7)	17·7 (13·1–23·2)	25·8 (20·3–29·6)	9·3 (7·8–11·2)	13·0 (3·9–23·7)
Singapore	34·0 (27·1–40·6)	17·8 (13·2–23·4)	25·9 (20·4–29·8)	9·3 (7·9–11·2)	13·0 (3·9–23·7)
High-income North America	33·3 (32·9–33·7)	18·0 (13·2–23·6)	26·2 (20·6–30·2)	9·4 (7·9–11·3)	13·1 (8·1–19·6)
Canada	34·9 (28·1–41·4)	17·6 (13·0–23·0)	25·5 (20·2–29·3)	9·2 (7·8–11·1)	12·8 (3·8–23·4)
Greenland	29·2 (21·6–36·5)	19·1 (13·9–25·0)	27·8 (21·8–32·2)	10·0 (8·4–11·9)	13·9 (4·2–25·3)
USA	33·2 (26·2–40·0)	18·0 (13·3–23·6)	26·2 (20·7–30·3)	9·4 (7·9–11·3)	13·2 (3·9–24·0)
Southern Latin America	29·6 (28·4–30·4)	19·0 (13·9–24·9)	27·6 (21·7–32·1)	9·9 (8·4–11·8)	13·9 (8·7–20·5)
Argentina	28·9 (21·4–36·4)	19·2 (14·0–25·1)	27·9 (22·0–32·5)	10·0 (8·5–11·9)	14·0 (4·2–25·4)
Chile	31·3 (24·4–38·4)	18·5 (13·6–24·3)	26·9 (21·1–31·2)	9·7 (8·2–11·6)	13·5 (4·1–24·5)
Uruguay	29·9 (22·4–37·2)	18·9 (13·8–24·7)	27·5 (21·7–31·8)	9·9 (8·3–11·8)	13·8 (4·1–25·0)
Western Europe	34·3 (33·9–34·8)	17·7 (13·1–23·2)	25·8 (20·3–29·7)	9·2 (7·8–11·2)	12·9 (7·9–19·4)
Andorra	34·7 (28·0–41·2)	17·6 (13·0–23·1)	25·6 (20·2–29·4)	9·2 (7·8–11·1)	12·9 (3·9–23·4)
Austria	34·4 (27·6–40·9)	17·7 (13·1–23·2)	25·8 (20·3–29·6)	9·2 (7·8–11·2)	12·9 (3·9–23·5)
Belgium	34·2 (27·4–40·8)	17·8 (13·2–23·3)	25·8 (20·3–29·7)	9·3 (7·8–11·2)	13·0 (3·9–23·6)
Cyprus	34·2 (27·3–40·8)	17·7 (13·1–23·3)	25·8 (20·3–29·7)	9·3 (7·8–11·2)	13·0 (3·9–23·6)
Denmark	34·0 (27·2–40·5)	17·8 (13·2–23·4)	25·9 (20·4–29·8)	9·3 (7·9–11·2)	13·0 (3·9–23·6)

Finland	34·4 (27·5–40·9)	17·7 (13·1–23·2)	25·8 (20·3–29·8)	9·2 (7·8–11·2)	12·9 (3·9–23·6)
France	34·4 (27·6–40·9)	17·7 (13·1–23·2)	25·7 (20·2–29·6)	9·2 (7·8–11·1)	12·9 (3·9–23·4)
Germany	34·2 (27·5–40·8)	17·7 (13·1–23·2)	25·8 (20·3–29·9)	9·3 (7·8–11·2)	13·0 (3·9–23·5)
Greece	33·7 (26·9–40·3)	17·9 (13·2–23·4)	26·0 (20·5–30·0)	9·3 (7·9–11·3)	13·1 (3·9–23·9)
Iceland	35·2 (28·4–41·6)	17·5 (13·0–22·9)	25·4 (20·0–29·2)	9·1 (7·7–11·1)	12·8 (3·8–23·3)
Ireland	34·8 (27·9–41·3)	17·6 (13·0–23·0)	25·6 (20·1–29·4)	9·2 (7·7–11·1)	12·8 (3·9–23·4)
Israel	33·5 (26·7–40·2)	17·9 (13·2–23·5)	26·1 (20·5–30·1)	9·4 (7·9–11·3)	13·1 (3·9–23·9)
Italy	34·8 (27·9–41·2)	17·6 (13·0–23·1)	25·6 (20·1–29·5)	9·2 (7·8–11·1)	12·9 (3·9–23·4)
Luxembourg	34·4 (27·5–41·0)	17·7 (13·1–23·2)	25·8 (20·3–29·6)	9·2 (7·8–11·2)	12·9 (3·9–23·5)
Malta	34·0 (27·2–40·6)	17·8 (13·1–23·3)	25·9 (20·4–29·8)	9·3 (7·8–11·2)	13·0 (3·9–23·6)
Monaco	34·4 (27·7–41·0)	17·7 (13·1–23·2)	25·7 (20·2–29·6)	9·2 (7·8–11·2)	12·9 (3·9–23·5)
Netherlands	34·9 (28·0–41·3)	17·6 (13·0–23·0)	25·5 (20·1–29·5)	9·2 (7·7–11·1)	12·8 (3·8–23·4)
Norway	34·8 (28·1–41·3)	17·6 (13·0–23·1)	25·6 (20·2–29·4)	9·2 (7·8–11·1)	12·9 (3·9–23·4)
Portugal	33·8 (27·0–40·3)	17·9 (13·1–23·4)	26·0 (20·5–29·8)	9·3 (7·9–11·2)	13·1 (3·9–23·7)
San Marino	34·6 (27·7–41·1)	17·6 (13·0–23·2)	25·7 (20·2–29·6)	9·2 (7·8–11·1)	12·9 (3·9–23·5)
Spain	34·8 (27·9–41·2)	17·6 (13·0–23·1)	25·6 (20·2–29·5)	9·2 (7·8–11·1)	12·9 (3·9–23·5)
Sweden	34·8 (27·9–41·3)	17·6 (13·0–23·1)	25·6 (20·2–29·4)	9·2 (7·8–11·1)	12·9 (3·9–23·4)
Switzerland	35·0 (28·3–41·6)	17·5 (12·9–23·0)	25·5 (20·1–29·3)	9·1 (7·7–11·0)	12·8 (3·8–23·3)
UK	33·7 (26·8–40·4)	17·9 (13·2–23·4)	26·0 (20·5–30·1)	9·3 (7·9–11·3)	13·1 (3·9–23·8)
Latin America and Caribbean	27·3 (25·2–28·4)	19·6 (14·2–25·7)	28·5 (22·5–33·3)	10·2 (8·6–12·2)	14·3 (9·1–21·1)
Andean Latin America	27·7 (25·7–28·7)	19·5 (14·2–25·6)	28·4 (22·3–33·2)	10·2 (8·6–12·1)	14·2 (9·1–21·1)
Bolivia	24·3 (15·1–32·7)	20·4 (14·7–26·8)	29·7 (23·4–35·1)	10·6 (9·0–12·7)	14·9 (4·4–27·0)
Ecuador	27·4 (19·2–35·2)	19·6 (14·3–25·7)	28·5 (22·5–33·3)	10·2 (8·6–12·2)	14·3 (4·3–25·9)
Peru	28·9 (21·5–36·4)	19·2 (14·0–25·2)	27·9 (21·9–32·4)	10·0 (8·5–11·9)	14·0 (4·2–25·4)
Caribbean	25·8 (22·9–27·2)	20·0 (14·5–26·2)	29·1 (22·9–34·4)	10·4 (8·8–12·4)	14·6 (9·4–21·5)
Antigua and Barbuda	28·5 (20·7–35·9)	19·3 (14·1–25·2)	28·1 (22·1–32·5)	10·1 (8·5–12·0)	14·1 (4·2–25·5)
The Bahamas	27·2 (19·2–34·9)	19·7 (14·3–25·8)	28·6 (22·5–33·5)	10·2 (8·7–12·2)	14·3 (4·3–26·0)
Barbados	28·7 (21·0–36·1)	19·2 (14·0–25·1)	28·0 (22·0–32·4)	10·0 (8·5–12·0)	14·0 (4·2–25·5)
Belize	26·3 (18·0–34·2)	19·9 (14·5–26·0)	28·9 (22·7–33·9)	10·4 (8·7–12·3)	14·5 (4·3–26·3)
Bermuda	32·6 (25·8–39·4)	18·2 (13·4–23·8)	26·4 (20·9–30·6)	9·5 (8·0–11·4)	13·3 (4·0–24·1)
Cuba	30·3 (23·1–37·4)	18·8 (13·8–24·7)	27·4 (21·6–31·7)	9·8 (8·3–11·7)	13·7 (4·1–24·9)
Dominica	25·6 (16·7–33·6)	20·1 (14·6–26·2)	29·2 (23·1–34·3)	10·5 (8·9–12·5)	14·6 (4·3–26·5)
Dominican Republic	25·7 (16·9–33·7)	20·1 (14·5–26·2)	29·2 (22·9–34·3)	10·5 (8·9–12·5)	14·6 (4·4–26·5)
Grenada	26·6 (18·4–34·5)	19·8 (14·4–25·9)	28·8 (22·7–34·0)	10·3 (8·7–12·3)	14·4 (4·3–26·1)
Guyana	23·5 (13·5–31·9)	20·7 (14·9–27·1)	30·0 (23·7–35·4)	10·8 (9·1–13·0)	15·1 (4·4–27·5)
Haiti	19·9 (8·1–29·5)	21·6 (15·4–28·1)	31·4 (24·7–37·3)	11·3 (9·5–13·5)	15·7 (4·6–28·7)
Jamaica	27·7 (19·7–35·5)	19·5 (14·2–25·6)	28·4 (22·4–33·0)	10·2 (8·6–12·1)	14·2 (4·3–25·7)
Puerto Rico	31·4 (24·4–38·4)	18·5 (13·5–24·3)	26·9 (21·1–31·1)	9·7 (8·2–11·5)	13·5 (4·0–24·6)
Saint Kitts and Nevis	27·0 (18·9–34·7)	19·7 (14·4–25·7)	28·7 (22·6–33·5)	10·3 (8·7–12·2)	14·4 (4·3–26·1)
Saint Lucia	27·2 (18·9–35·0)	19·7 (14·3–25·8)	28·6 (22·5–33·5)	10·2 (8·7–12·2)	14·3 (4·3–26·0)
Saint Vincent and the Grenadines	26·1 (17·7–34·2)	19·9 (14·4–26·1)	29·0 (22·8–34·1)	10·4 (8·8–12·4)	14·5 (4·3–26·3)
Suriname	24·9 (15·9–33·1)	20·3 (14·7–26·5)	29·5 (23·1–34·7)	10·6 (9·0–12·6)	14·8 (4·4–26·9)
Trinidad and Tobago	27·2 (19·2–35·1)	19·6 (14·2–25·6)	28·6 (22·5–33·4)	10·2 (8·6–12·2)	14·3 (4·3–26·0)
Virgin Islands	28·4 (20·6–36·0)	19·3 (14·1–25·3)	28·1 (22·1–32·6)	10·1 (8·5–12·0)	14·1 (4·2–25·6)
Central Latin America	27·5 (25·6–28·5)	19·6 (14·2–25·7)	28·4 (22·5–33·4)	10·2 (8·6–12·1)	14·3 (9·1–21·1)
Colombia	29·2 (21·5–36·5)	19·1 (13·9–24·9)	27·8 (21·8–32·3)	10·0 (8·4–11·9)	13·9 (4·2–25·4)
Costa Rica	30·1 (22·8–37·3)	18·9 (13·8–24·8)	27·5 (21·7–31·7)	9·8 (8·3–11·8)	13·8 (4·1–24·9)
El Salvador	27·9 (19·9–35·4)	19·5 (14·2–25·5)	28·3 (22·3–32·9)	10·1 (8·6–12·1)	14·2 (4·2–25·8)
Guatemala	25·0 (16·0–33·3)	20·3 (14·7–26·4)	29·5 (23·2–34·8)	10·6 (8·9–12·6)	14·8 (4·4–26·9)
Honduras	24·0 (14·7–32·5)	20·5 (14·8–26·7)	29·8 (23·4–35·1)	10·7 (9·0–12·8)	14·9 (4·4–27·2)
Mexico	27·4 (19·0–35·1)	19·6 (14·2–25·7)	28·5 (22·6–33·5)	10·2 (8·7–12·2)	14·3 (4·3–26·0)
Nicaragua	27·3 (19·2–35·1)	19·6 (14·2–25·7)	28·5 (22·5–33·5)	10·2 (8·6–12·2)	14·3 (4·3–25·9)
Panama	29·0 (21·4–36·3)	19·2 (13·9–25·2)	27·9 (21·9–32·4)	10·0 (8·5–11·9)	14·0 (4·2–25·4)
Venezuela	27·5 (19·3–35·2)	19·6 (14·2–25·7)	28·5 (22·5–33·3)	10·2 (8·7–12·1)	14·3 (4·3–25·8)
Tropical Latin America	27·3 (25·2–28·4)	19·6 (14·3–25·7)	28·5 (22·5–33·1)	10·2 (8·6–12·2)	14·3 (9·1–21·2)
Brazil	27·3 (19·1–35·1)	19·6 (14·3–25·7)	28·5 (22·5–33·1)	10·2 (8·6–12·2)	14·3 (4·3–25·8)
Paraguay	27·2 (18·9–35·0)	19·7 (14·3–25·7)	28·6 (22·5–33·3)	10·2 (8·7–12·2)	14·3 (4·3–25·9)
North Africa and Middle East	28·1 (26·4–29·0)	19·4 (14·1–25·4)	28·2 (22·3–33·0)	10·1 (8·6–12·1)	14·2 (9·0–21·0)
Afghanistan	21·4 (10·5–30·6)	21·2 (15·2–27·7)	30·8 (24·3–36·3)	11·1 (9·4–13·2)	15·5 (4·6–28·1)
Algeria	28·8 (21·2–36·1)	19·2 (14·0–25·1)	28·0 (21·9–32·5)	10·0 (8·5–11·9)	14·0 (4·2–25·5)
Bahrain	30·7 (23·4–37·7)	18·7 (13·7–24·5)	27·2 (21·5–31·5)	9·8 (8·3–11·7)	13·6 (4·1–24·9)
Egypt	27·2 (19·2–34·9)	19·6 (14·4–25·6)	28·6 (22·5–33·3)	10·2 (8·7–12·2)	14·3 (4·3–26·0)

Iran	29·9 (22·5–37·1)	18·9 (13·8–24·8)	27·5 (21·7–31·9)	9·9 (8·3–11·8)	13·8 (4·1–25·1)
Iraq	28·6 (21·1–36·2)	19·3 (14·0–25·2)	28·0 (22·0–32·7)	10·0 (8·5–11·9)	14·1 (4·2–25·4)
Jordan	30·1 (22·7–37·3)	18·9 (13·8–24·8)	27·4 (21·7–31·7)	9·8 (8·3–11·8)	13·8 (4·1–25·0)
Kuwait	32·4 (25·5–39·2)	18·2 (13·4–23·9)	26·5 (20·9–30·7)	9·5 (8·0–11·4)	13·3 (4·0–24·2)
Lebanon	31·1 (23·8–38·0)	18·6 (13·7–24·3)	27·1 (21·3–31·4)	9·7 (8·2–11·6)	13·6 (4·1–24·7)
Libya	28·7 (20·9–36·1)	19·3 (14·0–25·2)	28·0 (22·1–32·5)	10·0 (8·5–12·0)	14·0 (4·2–25·4)
Morocco	26·5 (18·0–34·4)	19·8 (14·4–25·9)	28·8 (22·7–33·9)	10·3 (8·7–12·3)	14·5 (4·3–26·2)
Oman	30·5 (23·4–37·7)	18·7 (13·7–24·6)	27·3 (21·5–31·5)	9·8 (8·3–11·7)	13·7 (4·1–24·7)
Palestine	28·6 (20·9–36·1)	19·3 (14·0–25·2)	28·0 (22·0–32·4)	10·0 (8·5–12·0)	14·1 (4·2–25·5)
Qatar	32·2 (25·3–39·1)	18·3 (13·4–24·0)	26·6 (21·0–30·7)	9·5 (8·1–11·5)	13·4 (4·0–24·3)
Saudi Arabia	29·9 (22·5–37·2)	18·9 (13·9–24·8)	27·5 (21·7–31·9)	9·9 (8·3–11·8)	13·8 (4·1–25·2)
Sudan	25·3 (16·5–33·5)	20·2 (14·6–26·4)	29·3 (23·2–34·4)	10·5 (8·9–12·5)	14·7 (4·4–26·6)
Syria	29·2 (21·6–36·5)	19·1 (14·0–25·0)	27·8 (21·9–32·4)	10·0 (8·4–11·9)	13·9 (4·2–25·4)
Tunisia	30·0 (22·6–37·1)	18·9 (13·8–24·8)	27·5 (21·7–31·8)	9·9 (8·3–11·8)	13·8 (4·1–25·1)
Türkiye	30·1 (22·7–37·4)	18·9 (13·7–24·6)	27·4 (21·7–31·8)	9·8 (8·3–11·7)	13·8 (4·1–24·9)
United Arab Emirates	28·7 (21·0–36·1)	19·2 (14·0–25·2)	28·0 (22·1–32·6)	10·0 (8·5–11·9)	14·0 (4·2–25·5)
Yemen	24·1 (14·4–32·6)	20·5 (14·8–26·9)	29·8 (23·5–35·2)	10·7 (9·1–12·8)	14·9 (4·4–27·3)
South Asia	24·1 (20·7–25·6)	20·5 (14·8–26·8)	29·8 (23·5–35·3)	10·7 (9·0–12·7)	14·9 (9·6–21·9)
Bangladesh	25·5 (16·8–33·7)	20·1 (14·5–26·3)	29·2 (23·0–34·2)	10·5 (8·9–12·5)	14·7 (4·4–26·6)
Bhutan	25·1 (16·2–33·3)	20·2 (14·6–26·4)	29·4 (23·1–34·6)	10·5 (8·9–12·6)	14·7 (4·4–26·8)
India	24·2 (14·7–32·6)	20·5 (14·8–26·8)	29·8 (23·5–35·3)	10·7 (9·0–12·7)	14·9 (4·4–27·0)
Nepal	24·2 (15·0–32·7)	20·5 (14·7–26·8)	29·7 (23·4–35·0)	10·7 (9·0–12·8)	14·9 (4·4–27·2)
Pakistan	22·5 (12·3–31·4)	20·9 (15·1–27·3)	30·4 (24·0–35·9)	10·9 (9·3–13·0)	15·2 (4·5–27·5)
Southeast Asia, east Asia, and Oceania	29·5 (28·3–30·2)	19·0 (13·9–24·9)	27·7 (21·8–32·1)	9·9 (8·4–11·9)	13·9 (8·7–20·6)
East Asia	31·3 (30·6–31·8)	18·5 (13·6–24·3)	27·0 (21·3–31·2)	9·7 (8·2–11·6)	13·5 (8·4–20·2)
China	31·4 (24·4–38·5)	18·5 (13·6–24·3)	26·9 (21·3–31·2)	9·7 (8·2–11·6)	13·5 (4·1–24·5)
North Korea	26·9 (18·7–34·7)	19·7 (14·4–25·8)	28·7 (22·6–33·7)	10·3 (8·7–12·2)	14·4 (4·3–26·1)
Taiwan (province of China)	32·7 (25·7–39·6)	18·2 (13·4–23·8)	26·4 (20·8–30·5)	9·5 (8·0–11·4)	13·3 (4·0–24·0)
Oceania	22·2 (17·6–24·2)	21·0 (15·1–27·4)	30·5 (24·0–36·2)	10·9 (9·3–13·1)	15·3 (9·9–22·4)
American Samoa	25·7 (17·0–33·8)	20·1 (14·6–26·2)	29·2 (23·0–34·3)	10·5 (8·8–12·5)	14·6 (4·4–26·6)
Cook Islands	29·8 (22·4–37·2)	18·9 (13·8–24·8)	27·5 (21·6–31·9)	9·9 (8·4–11·8)	13·8 (4·1–25·0)
Fiji	24·0 (14·6–32·5)	20·5 (14·7–26·9)	29·8 (23·4–35·3)	10·7 (9·0–12·8)	14·9 (4·4–27·2)
Guam	28·3 (20·6–36·0)	19·3 (14·0–25·2)	28·1 (22·2–32·7)	10·1 (8·5–12·0)	14·1 (4·2–25·6)
Kiribati	20·1 (8·3–29·6)	21·6 (15·4–28·1)	31·4 (24·7–37·1)	11·2 (9·5–13·5)	15·7 (4·6–28·7)
Marshall Islands	22·2 (11·8–31·1)	21·0 (15·1–27·5)	30·5 (24·0–36·1)	10·9 (9·3–13·1)	15·3 (4·5–27·8)
Federated States of Micronesia	23·2 (13·4–31·9)	20·8 (14·9–27·2)	30·2 (23·8–35·8)	10·8 (9·2–13·0)	15·1 (4·5–27·4)
Nauru	23·4 (13·7–32·0)	20·7 (14·9–27·1)	30·1 (23·6–35·5)	10·8 (9·1–12·9)	15·1 (4·5–27·2)
Niue	26·5 (18·3–34·3)	19·8 (14·4–26·0)	28·9 (22·7–33·9)	10·3 (8·7–12·3)	14·5 (4·3–26·3)
Northern Mariana Islands	28·1 (20·3–35·6)	19·4 (14·1–25·5)	28·2 (22·2–33·0)	10·1 (8·6–12·0)	14·2 (4·2–25·6)
Palau	26·5 (18·2–34·3)	19·9 (14·4–26·0)	28·9 (22·7–33·9)	10·3 (8·7–12·3)	14·5 (4·3–26·2)
Papua New Guinea	21·8 (11·1–31·0)	21·1 (15·1–27·5)	30·7 (24·1–36·3)	11·0 (9·4–13·2)	15·4 (4·5–28·1)
Samoa	25·3 (16·4–33·4)	20·2 (14·5–26·4)	29·3 (23·2–34·5)	10·5 (8·9–12·5)	14·7 (4·4–26·6)
Solomon Islands	21·7 (10·9–30·7)	21·2 (15·2–27·6)	30·7 (24·1–36·5)	11·0 (9·3–13·2)	15·4 (4·6–28·0)
Tokelau	25·9 (17·3–34·1)	20·0 (14·5–26·2)	29·1 (23·0–34·1)	10·4 (8·8–12·4)	14·6 (4·3–26·5)
Tonga	25·8 (17·1–33·8)	20·0 (14·6–26·3)	29·1 (23·0–34·2)	10·4 (8·9–12·5)	14·6 (4·3–26·5)
Tuvalu	23·8 (14·5–32·4)	20·6 (14·8–26·9)	29·9 (23·5–35·3)	10·7 (9·0–12·8)	15·0 (4·4–27·3)
Vanuatu	21·9 (11·3–30·9)	21·1 (15·1–27·6)	30·6 (24·0–36·2)	11·0 (9·3–13·2)	15·4 (4·5–28·0)
Southeast Asia	25·8 (23·3–27·1)	20·0 (14·5–26·2)	29·1 (22·9–34·2)	10·4 (8·8–12·4)	14·6 (9·3–21·5)
Cambodia	24·0 (14·3–32·4)	20·5 (14·8–26·8)	29·8 (23·5–35·3)	10·7 (9·0–12·7)	15·0 (4·4–27·1)
Indonesia	24·5 (15·3–32·9)	20·4 (14·7–26·7)	29·6 (23·3–34·9)	10·6 (9·0–12·6)	14·8 (4·4–26·9)
Laos	22·6 (12·7–31·5)	20·9 (15·0–27·3)	30·4 (23·9–35·8)	10·9 (9·2–13·0)	15·2 (4·5–27·7)
Malaysia	28·1 (20·2–35·6)	19·4 (14·2–25·3)	28·2 (22·2–32·8)	10·1 (8·5–12·1)	14·2 (4·2–25·7)
Maldives	29·2 (21·7–36·5)	19·1 (13·9–25·0)	27·8 (21·9–32·4)	10·0 (8·4–11·9)	13·9 (4·2–25·3)
Mauritius	28·2 (20·4–35·6)	19·4 (14·1–25·3)	28·2 (22·1–32·8)	10·1 (8·5–12·0)	14·1 (4·2–25·7)
Myanmar	23·9 (14·5–32·5)	20·6 (14·8–26·9)	29·9 (23·5–35·3)	10·7 (9·0–12·8)	15·0 (4·4–27·1)
Philippines	24·6 (15·5–32·8)	20·4 (14·7–26·7)	29·6 (23·5–34·8)	10·6 (9·0–12·6)	14·8 (4·4–26·8)
Seychelles	27·5 (19·6–35·2)	19·6 (14·2–25·6)	28·4 (22·5–33·3)	10·2 (8·6–12·1)	14·3 (4·2–25·9)
Sri Lanka	29·4 (21·7–36·7)	19·1 (13·9–25·1)	27·7 (21·9–32·1)	9·9 (8·4–11·9)	13·9 (4·2–25·3)
Thailand	29·7 (22·2–37·0)	19·0 (13·8–24·8)	27·6 (21·7–32·0)	9·9 (8·4–11·8)	13·8 (4·1–25·1)
Timor-Leste	23·1 (13·4–31·8)	20·8 (15·0–27·0)	30·2 (23·7–35·9)	10·8 (9·1–13·0)	15·1 (4·5–27·5)
Viet Nam	28·4 (20·6–35·9)	19·3 (14·1–25·3)	28·1 (22·1–32·7)	10·1 (8·5–12·0)	14·1 (4·2–25·5)
Sub-Saharan Africa	22·1 (17·7–24·1)	21·0 (15·1–27·4)	30·6 (24·1–36·2)	11·0 (9·3–13·1)	15·3 (9·9–22·4)

Central sub-Saharan Africa	21·4 (16·4–23·6)	21·2 (15·2–27·7)	30·9 (24·3–36·6)	11·1 (9·3–13·3)	15·5 (10·0–22·6)
Angola	21·8 (11·5–30·7)	21·1 (15·1–27·6)	30·7 (24·1–36·5)	11·0 (9·3–13·2)	15·4 (4·5–28·0)
Central African Republic	17·5 (4·6–27·7)	22·3 (15·8–29·1)	32·4 (25·4–38·9)	11·6 (9·8–14·0)	16·2 (4·8–29·6)
Congo (Brazzaville)	22·6 (12·5–31·4)	20·9 (15·0–27·3)	30·4 (23·9–36·0)	10·9 (9·2–13·1)	15·2 (4·5–27·8)
DR Congo	21·3 (10·5–30·7)	21·3 (15·2–27·7)	30·9 (24·4–36·6)	11·1 (9·4–13·4)	15·5 (4·6–28·3)
Equatorial Guinea	24·9 (16·1–33·1)	20·3 (14·7–26·5)	29·5 (23·1–34·7)	10·6 (8·9–12·5)	14·8 (4·4–26·7)
Gabon	24·2 (14·8–32·4)	20·5 (14·8–26·7)	29·8 (23·4–35·0)	10·7 (9·0–12·7)	14·9 (4·4–27·0)
Eastern sub-Saharan Africa	21·8 (17·2–23·9)	21·1 (15·1–27·6)	30·7 (24·2–36·3)	11·0 (9·3–13·2)	15·4 (10·0–22·5)
Burundi	20·3 (8·9–29·7)	21·5 (15·4–28·2)	31·3 (24·7–37·2)	11·2 (9·5–13·4)	15·7 (4·6–28·6)
Comoros	22·1 (12·0–31·1)	21·1 (15·1–27·6)	30·6 (24·1–36·1)	11·0 (9·3–13·2)	15·3 (4·5–27·9)
Djibouti	22·5 (12·3–31·4)	20·9 (15·0–27·4)	30·4 (24·0–36·0)	10·9 (9·3–13·1)	15·2 (4·5–27·9)
Eritrea	20·4 (9·5–29·8)	21·5 (15·4–28·1)	31·2 (24·7–37·0)	11·2 (9·5–13·5)	15·7 (4·6–28·7)
Ethiopia	22·2 (11·7–31·1)	21·0 (15·0–27·5)	30·5 (24·1–36·2)	10·9 (9·3–13·1)	15·3 (4·5–28·0)
Kenya	22·5 (12·4–31·3)	20·9 (15·0–27·3)	30·4 (23·9–35·9)	10·9 (9·2–13·0)	15·2 (4·5–27·8)
Madagascar	21·3 (10·4–30·6)	21·3 (15·2–27·7)	30·9 (24·3–36·6)	11·1 (9·4–13·3)	15·5 (4·6–28·2)
Malawi	21·7 (11·1–30·7)	21·1 (15·2–27·6)	30·7 (24·1–36·2)	11·0 (9·3–13·2)	15·4 (4·5–28·0)
Mozambique	20·4 (8·9–30·0)	21·5 (15·3–28·0)	31·2 (24·6–37·0)	11·2 (9·5–13·4)	15·6 (4·6–28·5)
Rwanda	22·2 (11·7–31·1)	21·0 (15·1–27·5)	30·5 (24·1–36·1)	10·9 (9·3–13·1)	15·3 (4·5–27·8)
Somalia	18·3 (5·7–28·2)	22·1 (15·7–28·8)	32·1 (25·3–38·4)	11·5 (9·7–13·9)	16·1 (4·7–29·4)
South Sudan	21·4 (10·3–30·6)	21·2 (15·2–27·7)	30·9 (24·4–36·4)	11·1 (9·4–13·2)	15·5 (4·6–28·2)
Uganda	22·4 (12·3–31·3)	21·0 (15·1–27·4)	30·5 (24·1–36·2)	10·9 (9·2–13·1)	15·3 (4·5–27·9)
Tanzania	22·5 (12·4–31·3)	20·9 (15·1–27·3)	30·4 (23·9–35·9)	10·9 (9·3–13·1)	15·2 (4·5–27·8)
Zambia	22·1 (11·8–31·0)	21·0 (15·1–27·5)	30·6 (24·0–36·2)	11·0 (9·3–13·0)	15·3 (4·5–27·8)
Southern sub-Saharan Africa	24·3 (20·9–25·9)	20·4 (14·7–26·7)	29·7 (23·4–35·1)	10·6 (9·0–12·7)	14·9 (9·6–21·8)
Botswana	23·7 (14·4–32·2)	20·6 (14·8–26·9)	29·9 (23·5–35·2)	10·7 (9·0–12·8)	15·0 (4·4–27·2)
Eswatini	22·5 (12·2–31·5)	20·9 (15·1–27·2)	30·4 (23·9–36·0)	10·9 (9·2–13·0)	15·2 (4·5–27·7)
Lesotho	20·7 (9·9–30·1)	21·4 (15·3–27·9)	31·1 (24·5–37·0)	11·2 (9·4–13·4)	15·6 (4·6–28·5)
Namibia	24·2 (15·1–32·7)	20·5 (14·7–26·7)	29·7 (23·4–35·1)	10·7 (9·0–12·7)	14·9 (4·4–27·0)
South Africa	25·3 (16·3–33·5)	20·2 (14·5–26·3)	29·3 (23·1–34·4)	10·5 (8·9–12·5)	14·7 (4·4–26·6)
Zimbabwe	21·3 (10·3–30·5)	21·3 (15·2–27·8)	30·9 (24·1–36·6)	11·1 (9·4–13·2)	15·5 (4·6–28·2)
Western sub-Saharan Africa	22·2 (17·9–24·2)	21·0 (15·1–27·4)	30·5 (24·0–36·1)	10·9 (9·3–13·1)	15·3 (9·9–22·4)
Benin	22·0 (11·5–31·0)	21·1 (15·1–27·5)	30·6 (24·0–36·2)	11·0 (9·3–13·1)	15·3 (4·5–27·9)
Burkina Faso	21·3 (10·7–30·4)	21·3 (15·2–27·8)	30·9 (24·3–36·5)	11·1 (9·4–13·3)	15·5 (4·6–28·1)
Cabo Verde	26·8 (18·5–34·6)	19·8 (14·3–25·8)	28·7 (22·7–33·6)	10·3 (8·7–12·3)	14·4 (4·3–26·1)
Cameroon	22·6 (12·2–31·4)	20·9 (15·0–27·3)	30·4 (24·0–35·9)	10·9 (9·2–13·0)	15·2 (4·5–27·7)
Chad	20·1 (8·7–29·5)	21·6 (15·4–28·2)	31·4 (24·7–37·2)	11·2 (9·5–13·5)	15·7 (4·6–28·6)
Côte d'Ivoire	22·7 (12·9–31·5)	20·9 (15·0–27·3)	30·3 (23·9–35·6)	10·9 (9·2–13·0)	15·2 (4·5–27·7)
The Gambia	22·9 (12·8–31·6)	20·8 (15·0–27·2)	30·3 (23·8–35·9)	10·9 (9·2–12·9)	15·2 (4·5–27·5)
Ghana	23·4 (13·6–32·0)	20·7 (14·9–26·9)	30·1 (23·6–35·3)	10·8 (9·1–12·8)	15·1 (4·5–27·3)
Guinea	20·8 (10·1–30·1)	21·4 (15·3–28·0)	31·1 (24·6–36·9)	11·1 (9·4–13·4)	15·6 (4·6–28·5)
Guinea-Bissau	20·1 (8·9–29·4)	21·6 (15·5–28·1)	31·4 (24·7–37·1)	11·2 (9·5–13·6)	15·7 (4·7–28·6)
Liberia	22·9 (12·9–31·6)	20·8 (15·0–27·3)	30·3 (23·8–35·8)	10·8 (9·2–13·0)	15·2 (4·5–27·6)
Mali	21·6 (11·1–30·8)	21·2 (15·1–27·7)	30·8 (24·0–36·5)	11·0 (9·3–13·2)	15·4 (4·5–28·1)
Mauritania	24·6 (15·5–33·0)	20·4 (14·7–26·6)	29·6 (23·3–35·0)	10·6 (9·0–12·6)	14·8 (4·4–26·8)
Niger	20·7 (9·3–30·0)	21·4 (15·3–28·1)	31·1 (24·4–36·8)	11·2 (9·4–13·4)	15·6 (4·6–28·5)
Nigeria	22·5 (12·3–31·3)	20·9 (15·1–27·2)	30·4 (23·9–36·0)	10·9 (9·3–13·0)	15·3 (4·5–27·7)
São Tomé and Príncipe	24·8 (15·7–33·0)	20·3 (14·7–26·6)	29·5 (23·2–34·8)	10·6 (8·9–12·6)	14·8 (4·4–26·8)
Senegal	22·8 (12·8–31·6)	20·9 (15·0–27·2)	30·3 (24·0–35·8)	10·9 (9·2–13·0)	15·2 (4·5–27·6)
Sierra Leone	21·9 (11·5–30·9)	21·1 (15·1–27·5)	30·7 (24·2–36·4)	11·0 (9·3–13·1)	15·4 (4·5–27·9)
Togo	22·5 (12·3–31·4)	20·9 (15·0–27·3)	30·4 (23·8–35·9)	10·9 (9·2–13·0)	15·2 (4·5–27·7)

Table S8. PRISMA Checklist

Section and Topic	Item #	Checklist item	Location where item is reported
**TITLE**			
Title	1	Identify the report as a systematic review.	N/A, contains meta-analysis
**ABSTRACT**			
Abstract	2	See the PRISMA 2020 for Abstracts checklist.	
**INTRODUCTION**			
Rationale	3	Describe the rationale for the review in the context of existing knowledge.	Research in context, Method-Treatment effect estimation(pages 3, 5)
Objectives	4	Provide an explicit statement of the objective(s) or question(s) the review addresses.	Overview, Method (pages 5-6)
**METHODS**			
Eligibility criteria	5	Specify the inclusion and exclusion criteria for the review and how studies were grouped for the syntheses.	Method-Treatment effect estimation (page 5, appendix page 8)
Information sources	6	Specify all databases, registers, websites, organisations, reference lists and other sources searched or consulted to identify studies. Specify the date when each source was last searched or consulted.	Method-Treatment effect estimation, Figure S5 (page 5, appendix page 8)
Search strategy	7	Present the full search strategies for all databases, registers and websites, including any filters and limits used.	Method-Treatment effect estimation, Figure S5 (page 5, appendix page 8)
Selection process	8	Specify the methods used to decide whether a study met the inclusion criteria of the review, including how many reviewers screened each record and each report retrieved, whether they worked independently, and if applicable, details of automation tools used in the process.	Method-Treatment effect estimation, Figure S5 (page 5, appendix page 8)
Data collection process	9	Specify the methods used to collect data from reports, including how many reviewers collected data from each report, whether they worked independently, any processes for obtaining or confirming data from study investigators, and if applicable, details of automation tools used in the process.	Method, page 5
Data items	10a	List and define all outcomes for which data were sought. Specify whether all results that were compatible with each outcome domain in each study were sought (e.g. for all measures, time points, analyses), and if not, the methods used to decide which results to collect.	Method, page 5
	10b	List and define all other variables for which data were sought (e.g. participant and intervention characteristics,	Method, page 5,

		funding sources). Describe any assumptions made about any missing or unclear information.	Appendix page 8<br>Network Meta-analysis comparison counts and Appendix Table S4
Study risk of bias assessment	11	Specify the methods used to assess risk of bias in the included studies, including details of the tool(s) used, how many reviewers assessed each study and whether they worked independently, and if applicable, details of automation tools used in the process.	N/A
Effect measures	12	Specify for each outcome the effect measure(s) (e.g. risk ratio, mean difference) used in the synthesis or presentation of results.	Method-Treatment effect estimation, standardized mean difference (SMD), page 6
Synthesis methods	13a	Describe the processes used to decide which studies were eligible for each synthesis (e.g. tabulating the study intervention characteristics and comparing against the planned groups for each synthesis (item #5)).	Method-Treatment effect estimation, page 5-6
	13b	Describe any methods required to prepare the data for presentation or synthesis, such as handling of missing summary statistics, or data conversions.	No missing data because the results were from Cochrane reviews.
	13c	Describe any methods used to tabulate or visually display results of individual studies and syntheses.	Method-Treatment effect estimation, page 5-6, through MR-BRT
	13d	Describe any methods used to synthesize results and provide a rationale for the choice(s). If meta-analysis was performed, describe the model(s), method(s) to identify the presence and extent of statistical heterogeneity, and software package(s) used.	Method-Treatment effect estimation, page 5-6, R package escalc and MR-BRT were used
	13e	Describe any methods used to explore possible causes of heterogeneity among study results (e.g. subgroup analysis, meta-regression).	Method-Treatment effect estimation, page 5-6, gamma was calculated
	13f	Describe any sensitivity analyses conducted to assess robustness of the synthesized results.	N/A
Reporting bias assessment	14	Describe any methods used to assess risk of bias due to missing results in a synthesis (arising from reporting biases).	Method-Treatment effect estimation, page 5-6, gamma was calculated
Certainty assessment	15	Describe any methods used to assess certainty (or confidence) in the body of evidence for an outcome.	All the analysis were repeated 1000 times for estimation.
**RESULTS**			

Study selection	16a	Describe the results of the search and selection process, from the number of records identified in the search to the number of studies included in the review, ideally using a flow diagram.	Results, Table 1, page 10, Appendix, Figure S5
	16b	Cite studies that might appear to meet the inclusion criteria, but which were excluded, and explain why they were excluded.	All listed in the references, pages 15-19, appendix, Figure S5
Study characteristics	17	Cite each included study and present its characteristics.	Results, Table 1, page 10, Appendix, Figure S5, extraction sheet is available on github
Risk of bias in studies	18	Present assessments of risk of bias for each included study.	Method-Treatment effect estimation, page 5-6, gamma was calculated
Results of individual studies	19	For all outcomes, present, for each study: (a) summary statistics for each group (where appropriate) and (b) an effect estimate and its precision (e.g. confidence/credible interval), ideally using structured tables or plots.	Results, Table 1, page 10, all analyses contains 95% CI and were repeated 1000 times
Results of syntheses	20a	For each synthesis, briefly summarise the characteristics and risk of bias among contributing studies.	Results, Table 1,2, Figure 1, pages 8-12
	20b	Present results of all statistical syntheses conducted. If meta-analysis was done, present for each the summary estimate and its precision (e.g. confidence/credible interval) and measures of statistical heterogeneity. If comparing groups, describe the direction of the effect.	Results, Table 1, page 10
	20c	Present results of all investigations of possible causes of heterogeneity among study results.	Method-Treatment effect estimation, page 5-6, gamma was calculated, Results, page 10
	20d	Present results of all sensitivity analyses conducted to assess the robustness of the synthesized results.	Results, Table 1, page 10
Reporting biases	21	Present assessments of risk of bias due to missing results (arising from reporting biases) for each synthesis assessed.	No missing data in the data preparation.
Certainty of evidence	22	Present assessments of certainty (or confidence) in the body of evidence for each outcome assessed.	Methods, results, discussion. Results, Table 1, page 10, all analyses contain 95% CI and were repeated 1000 times

**DISCUSSION**			
Discussion	23a	Provide a general interpretation of the results in the context of other evidence.	Discussion, pages 12-14
	23b	Discuss any limitations of the evidence included in the review.	Discussion, pages 12-14
	23c	Discuss any limitations of the review processes used.	Discussion, page 14
	23d	Discuss implications of the results for practice, policy, and future research.	Discussion, pages 14-15
**OTHER INFORMATION**			
Registration and protocol	24a	Provide registration information for the review, including register name and registration number, or state that the review was not registered.	NA, secondary analysis
	24b	Indicate where the review protocol can be accessed, or state that a protocol was not prepared.	The data and the code are available on github
	24c	Describe and explain any amendments to information provided at registration or in the protocol.	Secondary analysis, no registration
Support	25	Describe sources of financial or non-financial support for the review, and the role of the funders or sponsors in the review.	Pages, 2, 15
Competing interests	26	Declare any competing interests of review authors.	Page 15
Availability of data, code and other materials	27	Report which of the following are publicly available and where they can be found: template data collection forms; data extracted from included studies; data used for all analyses; analytic code; any other materials used in the review.	Data extraction and code are available on git

292

References

1. Burstein R, Fleming T, Haagsma J, Salomon JA, Vos T, Murray CJ. Estimating distributions of health state severity for the global burden of disease study. *Popul Health Metr* 2015; **13**: 31.
2. Zheng P, Barber R, Sorensen RJD, Murray CJL, Aravkin AY. Trimmed Constrained Mixed Effects Models: Formulations and Algorithms. *Journal of Computational and Graphical Statistics* 2021; **30**(3): 544-56.
3. Quality AfHRa. Medical Expenditure Panel Survey (MEPS). 2021. https://www.meps.ahrq.gov/mepsweb/ (accessed September 6 2022).
4. Blewett. Lynn. RDJ, Griffin. KC., Williams. KC. . IPUMS Health Surveys: Medical Expenditure Panel Survey,. In: IUPUMS, editor. 11 ed; 2019.
5. Fullman N, Yearwood J, Abay SM, et al. Measuring performance on the Healthcare Access and Quality Index for 195 countries and territories and selected subnational locations: a systematic analysis from the Global Burden of Disease Study 2016. *The Lancet* 2018; **391**(10136): 2236-71.
